# Multiple genetic variants at the *SLC30A8* locus affect a local super-enhancer cluster to influence pancreatic β-cell survival and function

**DOI:** 10.1101/2023.07.13.548906

**Published:** 2023-07-13

**Authors:** Ming Hu, Silvia Bonas-Guarch, Innah Kim, Ignasi Morán, Weicong Peng, Amélie Bonnefond, Amna Khamis, Philippe Froguel, Guy A. Rutter

**Affiliations:** 1 Section of Cell Biology and Functional Genomics, Division of Diabetes, Endocrinology and Metabolism, Department of Metabolism, Digestion and Reproduction, Faculty of Medicine, Imperial College London, Du Cane Road, London W12 0NN, UK; 2 Department of Metabolism, Digestion, and Reproduction, Imperial College London, Hammersmith Hospital Campus, Du Cane Road, London W12 0NN, UK; 3 Center for Genomic Regulation (CRG), C/ Dr. Aiguader, 88, PRBB Building, 08003 Barcelona, Spain; 4 Life Sciences Department, Barcelona Supercomputing Center (BSC-CNS), 08034 Barcelona, Spain; 5 Inserm U1283, CNRS UMR 8199, EGID, Institut Pasteur de Lille, F-59000, France.; 6 University of Lille, Lille University Hospital, Lille, F-59000, France.France; 7 Centre de Recherche du CHUM, Faculté de Médicine, Université de Montréal, Montréal, QC, Canada, Lee Kong Chian Imperial Medical School, Nanyang Technological University, Singapore

## Abstract

Variants at the *SLC30A8* locus are associated with type 2 diabetes (T2D) risk. The lead variant, rs13266634, encodes an amino acid change, Arg325Trp (R325W), at the C-terminus of the secretory granule-enriched zinc transporter, ZnT8. Although this protein-coding variant was previously thought to be the sole driver of altered disease risk, recent studies have provided evidence for lowered expression of *SLC30A8* mRNA in protective allele carriers. In the present study, combined allele-specific expression (cASE) analysis in human islets revealed that multiple variants affect the expression *SLC30A8*. Chromatin accessibility and epigenomic analyses imply the existence at the *SLC30A8* locus of an islet-selective super-enhancer cluster hosting multiple diabetes-associated variants. The variant region is spatially associated with both the *SLC30A8* promoter and with the regulatory regions of the neighbouring *RAD21, RAD21-AS1, UTP23* and other genes. Deletion of variant-bearing regions from human-derived EndoC-βH3 cells using CRISPR-Cas9 lowered the expression of *SLC30A8* and several neighbouring genes, suggesting their co-regulation by this enhancer cluster. Whilst deletion of *SLC30A8* had no effect on beta cell survival under the conditions examined, loss of *RAD21 or UTP23* markedly reduced cell viability. Thus, the protective effects of variants that lower *SLC30A8* activity may be modulated by the altered expression of nearby genes. Direct evidence for this possibility was not, however, obtained by cASE or eQTL analysis of human islet samples.

## Introduction

Type 2 Diabetes (T2D) affects more than 400 million people worldwide and the total number of people affected by diabetes is expected to increase to >600 million by 2045 (https://www.diabetesatlas.org). While cultural and environmental factors are important elements in disease risk, genetics also plays a major role. Thus, more than 200 genetic loci carrying >400 independent signals associated with T2D have been discovered [1–4], with the majority of these influencing insulin secretion [5–7]. Analyses of chromatin accessibility and expression quantitative trait (eQTL) loci in human islets have further increased our understanding of how variants are likely to exert their effects [8, 9]. These, alongside functional studies to determine the role of implicated gene products in pancreatic β cell function, have allowed us [10–13] to determine the likely mode(s) of action of variants at several disease-associated *loci*.

The *SLC30A8* locus was one of the first to be identified by genome-wide association studies (GWAS) as being associated with T2D [6] and has attracted considerable interest ever since [14, 15]. The lead single nucleotide polymorphism (SNP) rs13266634 is located in exon 8 of *SLC30A8*, a gene expressed almost exclusively in the endocrine pancreas. Rs13266634 is a non-synonymous variant that alters the primary sequence of the secretory granule zinc transporter ZnT8 (SLC30A8) at the C-terminus of the protein, likely affecting monomer dimerization [16–18], and thus intrinsic Zn^2+^ transporter activity. Nevertheless, the mechanisms through which this change may affect disease risk remain unclear. Firstly, structural predictions and functional studies exploring the impact of the common R325W variant on ZnT8 have been inconclusive. Evidence for both diminished [16] and increased [18] intrinsic zinc transporter activity of the risk (R325) variant has been obtained using different approaches.

Extensive studies examining the impact of deletion or inactivation of *SLC30A8* in humans, mice and in cell lines have yielded contradictory results. Thus, several [16, 19], though not all [14, 20], studies in mice have demonstrated a requirement for *Slc30a8* for normal insulin granule biogenesis and Zn^2+^ accumulation, and for normal insulin secretion *in vivo* and/or *in vitro*. In contrast, rare loss-of-function *SLC30A8* variants appear to be beneficial for human β-cell function, lowering T2D risk [21, 22]. Correspondingly, the deletion of *SLC30A8* from human beta cells was found to enhance insulin secretion [22], whilst the p.Arg138* loss of function (LoF) variant protected against apoptosis at low extracellular Zn^2+^ concentrations [23].

GWAS studies [22] have also identified a cluster of non-coding genetic variants at *SLC30A8* locus (https://t2d.hugeamp.org) [26], whose contribution to disease risk relative to rs13266634 is unclear. Our recent findings [27] have suggested that carriers of protective alleles at this locus display lowered *SLC30A8* transcript levels. Whether these actions are due to altered chromatin accessibility at this locus, and hence altered transcription, or are mediated by post-transcriptional events such as altered mRNA stability, has not been assessed. Likewise, the possible contribution of other genes, which may be co-ordinately regulated with *SLC30A8* at this locus, has not been explored.

Here, we sought to determine whether rs13266634 or other variants might affect the transcription of *SLC30A8* or other nearby genes, with consequences for β cell function or survival.

## Results

### Impact of T2D variants on transcript levels in human islets

GWAS-identified variants at the 3’ end of *SLC30A8* are shown in Figure S1 (www.t2d.hugeamp.org). To determine whether the variants were associated with the expression of the *SLC30A8* gene as well as nearby genes in human islets, we first performed expression quantitative trail *loci* (eQTL) analyses using data from multiple sources. As shown in Table 1A–1E, no statistically significant associations were identified in the cohorts examined.

**Figure 1.**
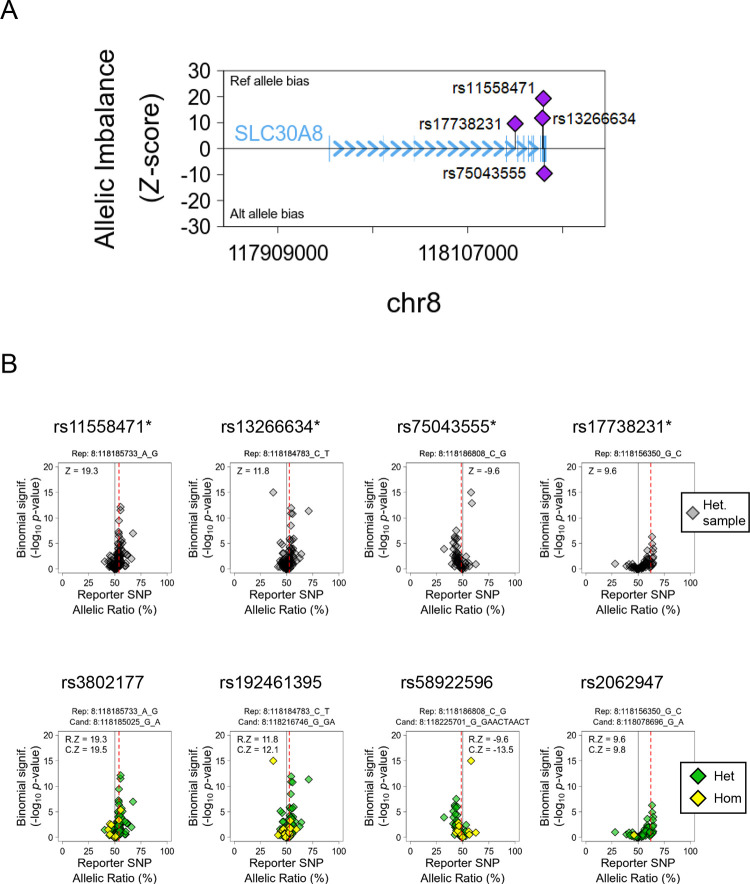
Combined Allele-Specific Expression (cASE) Analysis at the *SLC30A8* locus. A. Location of the reporter variants with significant cASE signal. The y-axis shows the strength and direction of their effect (Z-score). B. Detail of *SLC30A8* reporter variants (top panel) and their top candidate cis-regulatory variant to drive the cASE effect (bottom panel). The samples are grouped by the genotype of the candidate variant, heterozygous (Het., green), and homozygous (Hom., yellow). The first group needs to have a significant cASE for the gene (C.Z), while the second need to show no significant cASE. If C.Z is stronger than the reporter cASE calculated with all the samples (R.Z), the variant is considered a candidate.

Given the absence of eQTL associations we turned next to combined allele-specific expression (cASE) analyses, interrogating published RNA-seq data from heterozygous islet samples [27]. Because both alleles in heterozygotes are exposed to the same milieu, this approach circumvents confounding effects of non-genetic variables (cold ischemia time, donor health and demographic variables, pre-mortem therapies, etc). Figure 1A illustrates the location of the four significant cASE reporter variants in the *SLC30A8* gene.

We extended our earlier cASE analysis at this locus [27] demonstrating highly correlated rs11558471 and rs13266634 variants (LD r^2^ = 0.96) exhibited a strong allelic bias of *SLC30A8* expression (*p*<4.64 × 10^−14^ and *p*<2.88 × 10^−6^ respectively) (Figure 1B, upper panel). The rs13266634 T2D risk allele (C) is the preferential allele for transcription. Additionally, we identified two independent cASE signals, rs17738231 (*p*<1.06 × 10^−4^) and rs75043555 (*p*<1.13 × 10^−4^), which have no known GWAS associations (Table 2). Other associations are reported in Figure 1B (bottom panel). In sum, these data indicate that the allele-biased expression of *SLC30A8* overlapped with previous GWAS signals, and identified independent non-GWAS variants that may also play a significant role in the transcriptional regulation of the gene.

Using the same strategy, we performed cASE analysis to detect differential expression of nearby genes. No significant islet cASE associations were apparent for *RAD21, MED30*, EXT1, *UTP23* or *RP11–654G14.1. RAD21-AS1* could not be examined due to the absence of suitable variants in the transcript (data not shown). Overall, these data suggest that the variants located in the region are associated with allele specific expression of *SLC30A8* but not of nearby genes.

**Figure 2.**
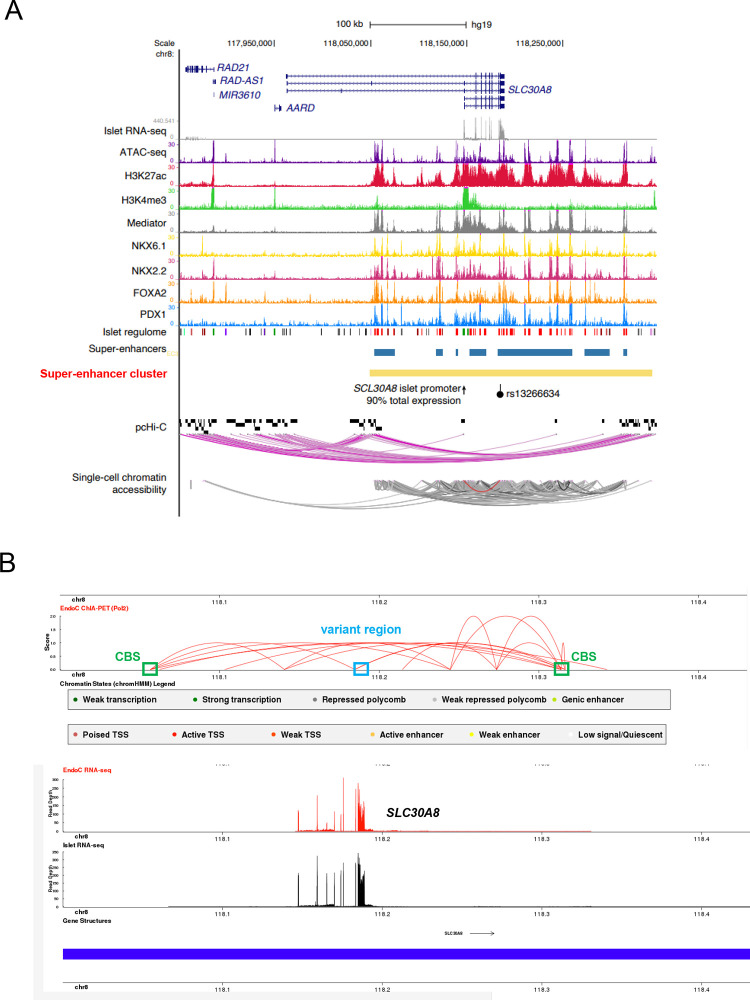
Combined epigenetic and chromatin maps at the *SLC30A8* locus. A. Integrative map of the *SLC30A8* locus in human islets showing chromatin state profiles and chromatin interactions between the high occupancy enhancer cluster (in yellow) and *SLC30A8* and nearby genes, as determined by pcHi-C and single-cell chromatin accessibility analysis. The lead T2D GWAS variant rs13266634 is represented as a black dot. B. CHIA-PET map [50] of the *SLC30A8* locus showing spatial contacts between the variant region and nearby regions. Polymerase II antibody was used to pull down protein/genomic DNA complex followed by whole genome sequencing [50]. Blue box: the variant region. Green box: CTCF binding sites (CBS) representing the 5’ and 3’ end of the super-enhancer cluster. Note that the variant region is spatially associated with the CTCF binding sites of the enhancer cluster.

**Figure 3.**
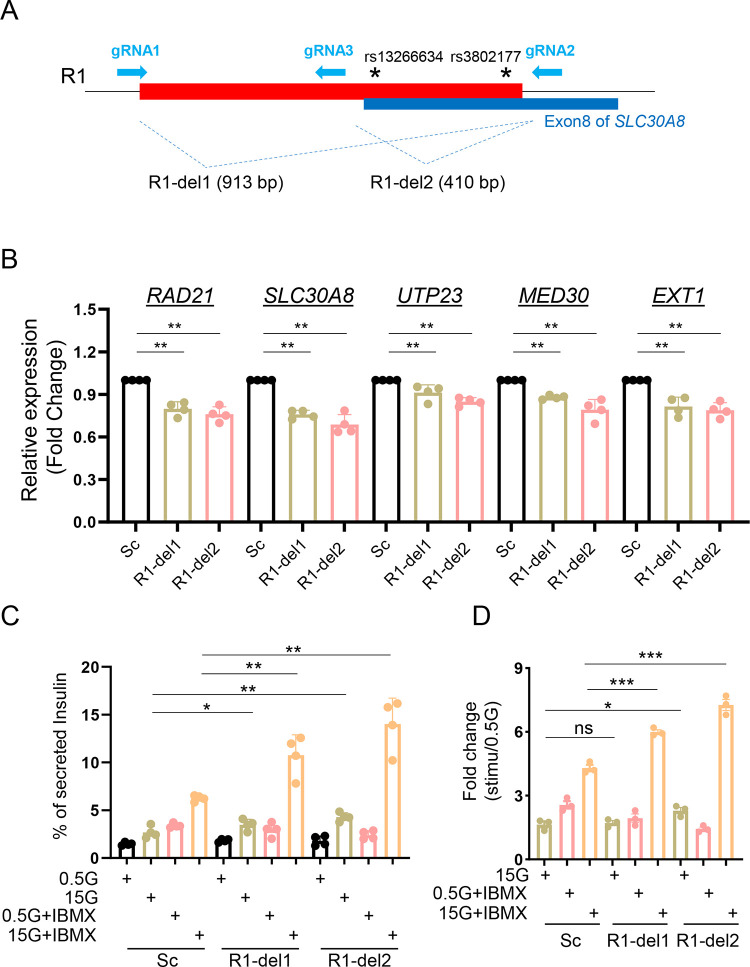
Role of R1 enhancer region in β-cell function. A. Diagram of R1 enhancer deletion in EndoC-βH3 by CRISPR-Cas9 mediated genome editing. Two gRNAs were designed to delete either entire R1 region (R1-del1 by gRNA1 and gRNA2) or a smaller region containing two variants (R1-del2 by gRNA3 and gRNA2). In both cases, deletions resulted into the loss of exon 8 of the *SLC30A8* gene, thus may affect ZnT8 translation. B. Taqman^™^ qRT-PCR analysis of gene expression in R1-del cells. C. Glucose stimulated insulin secretion (GSIS) assay. Data are mean ± SEM. *, *P* < 0.05; **, *P* < 0.01; ***, *P* < 0.005. . D. Fold change of secreted insulin. Data were normalized to insulin secretion at basal level (0.5 mM glucose); *n* = 3.

To explore the molecular mechanisms that elicit allele-specific expression of *SLC30A8,* and to determine whether altered transcription was likely to contribute to disease risk, we turned to local epigenomic maps of human islets (http://epigenomegateway.wustl.edu/browser/). These maps identified a high occupancy cluster of active super-enhancers [28] (Figure 2A, S2A and S3A). This cluster, stretching about 280 kb (Figure S2A), is characterized by high levels of histone-H3 lysine-27 acetylation (H3K27ac), binding of transcriptional regulator CCCTC binding factor CTCF, and cohesin complex (Figure 3A), as well as multiple islet specific-transcription factors (Figure 2A and S3B). Analyses of epigenomic maps in other human subjects (http://compbio2.mit.edu/epimap_vis/) [29] suggested that this enhancer cluster is chiefly active in pancreatic islets (Figure S2A–S2E). Accordingly, *SLC30A8* mRNA is almost exclusively expressed in islets in man (https://tiger.bsc.es) [27] and in other mammals [16, 19].

Hi-CHIP [30], Promoter-HiC [5] and CHIA-pet [31] analyses of human islet samples demonstrated that a group of genes at the 5’ end of the *SLC30A8* locus, including *RAD21* and *RAD21-AS1,* is spatially associated with the enhancer cluster through chromatin loop formation (Figure 2A and S3A). The variant region, which is embedded within the super-enhancer cluster, is also spatially associated with the promoter regions through single-cell chromatin accessibility analysis, suggesting that these transcripts may be co-regulated by the variant-bearing enhancers (Figure 2A). These maps indicate that within the same topologically associating domain (TAD), the super-enhancer cluster and embedded GWAS variants might influence the expression levels of not only *SLC30A8* but also nearby genes including *UTP23*, *RAD21*, *MED30* and *EXT1* and two long noncoding RNAs *RAD21-AS1* and *RP11–654G14.1.*

Several of the above genes encode components of complexes involved in fundamental cellular processes. *RAD21* is a cleavable component of the cohesin complex, involved in chromosome segregation, DNA replication, and other events [32]. The cohesin complex forms a ring structure that allows DNA to be extruded, controlling DNA looping. *UTP23* is a component of the small subunit processome (SSU) involved in rRNA-processing, ribosome biogenesis and 18S rRNA maturation [33]. *MED30* (mediator of RNA polymerase II transcriptional subunit 30) is part of the mediator complex bridging the enhancers and promoters [34]. *EXT1*, which encodes Exostosin-1 protein, is one of the two endoplasmic reticulum-resident type II transmembrane glycosyltransferase. Loss of function mutation in *EXT1* affects β-cell mass and insulin secretion in humans [35]. *RAD21-AS1* is expressed in the opposite direction to *RAD21*, while *RP11–654G14.1* is located in an intron within *SLC30A8*. Their roles in β-cells are unknown, and may include actions as enhancer RNAs (eRNA) [36].

### T2D GWAS variants are located in the SLC30A8 super-enhancer cluster

Exploration of the credible set at this locus indicated that rs13266634 carries the highest posterior probability for disease risk (Table 3). To determine whether any of the other variants may drive altered gene expression we performed a functional screen to assess their activity in human EndoC-βH3 cells. Five out of seven GWAS-identified variants were selected for these studies (Figure S1). Three variants (rs13266634, rs3802177, rs35859536) lie within two enhancer regions (termed R1, R2) with islet enhancer elements (enriched ATAC-seq, H3K27ac signals and islet-specific transcriptional factor bindings in the epigenomic maps) (Figure S3A and S3B). Both R1 and R2 regions are located at the 3’ end of the *SLC30A8* locus and are physically close to the CTCF binding sites of the islet-enriched enhance cluster (“CBS”) as assessed by CHIA-PET mapping (Figure 2B)[30]. R1 overlaps with exon 8 of *SLC30A8* and hosts two variants, rs13266634, in the protein coding region, and rs3802177 in the 3’ UTR. *In silico* analysis of transcription factor binding using JASPAR [37] suggested differential transcription factor binding (Table 4) between risk and protective variants. Two variants (rs3802177 and rs11558471) are located in the 3’ UTR of *SLC30A8* and may thus affect *SLC30A8* mRNA stability. *In silico* analysis suggests altered miRNA binding between the risk and protective variants (Table 4).

R1 and R2 are deeply embedded in one of seven super-enhancers (Figure 2A). To assess whether R1 exhibits transcriptional activity, we cloned different lengths of the genomic DNA of R1 region into pGL4.32 reporter vector which carries mini cytomegalovirus (CMV) promoter, and performed promoter-luciferase assays in EndoC-βH3 cells (Figure S3A–S3C). Luciferase activities of all DNA fragments were lower than empty vector indicating that this region is unlikely to have intrinsic enhancer activity, but may influence the activity of neighbouring enhancers.

To explore these possibilities in the context of genomic DNA, we deleted R1 from EndoC-βH3 cells through CRISPR-Cas9-mediated genome editing [13]. Three gRNAs were designed (Figure 3A) to delete: (1) the entire R1 region (R1-del1) or (2), a small R1 region bearing two GWAS variants (R1-del2). Genomic DNA deletions were confirmed by Sanger sequencing (Figure S4D and S4E). The deletion efficiency was around 50–60% based on the protocol developed in our previous report (Figure S4F and S4G) [13]. Compared to control cells infected with scrambled gRNA sequences both R1-del1 and R1-del2 cells displayed lower expression of *SLC30A8* as well as several nearby genes (Figure 3B). Both R1-del1 and R1-del2 cells displayed increased insulin secretion stimulated by high glucose (15 vs 0.5 mM) and the phosphodiesterase inhibitor isobutylmethyl xanthine (IBMX; 0.5mM) (Figure 3C and 3D). Thus, lowered transcriptional activity brought about by R1-deletion improves β cell function.

**Figure 4.**
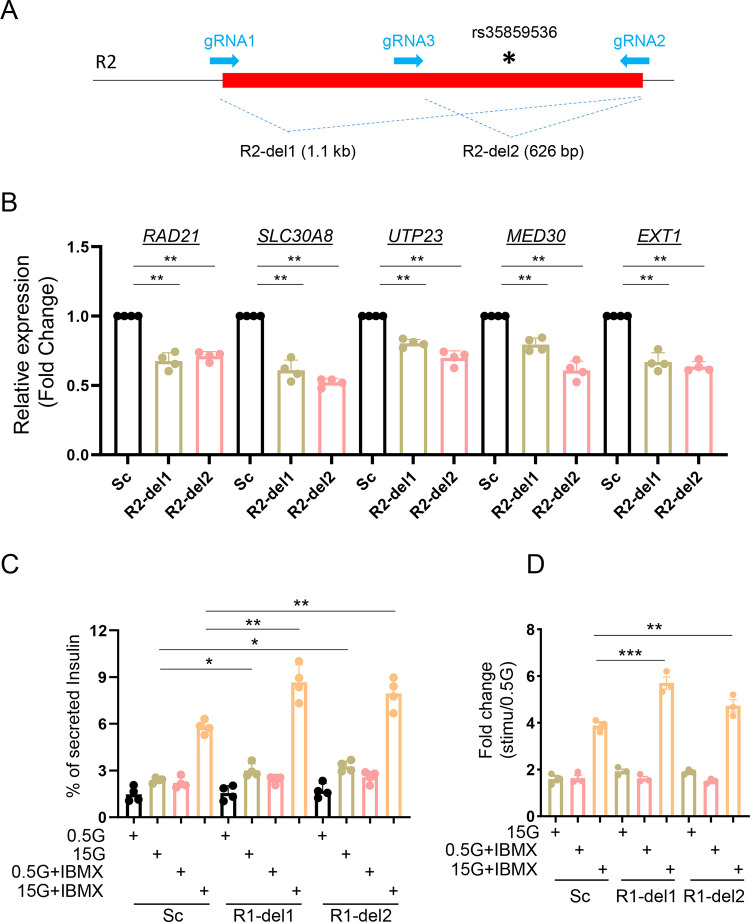
Role of R2 region in β-cell function. A. Diagram of R2 deletion in EndoC-βH3 by CRISPR-Cas9 mediated genome editing. Two gRNAs were designed to delete entire R2 region (R2-del1 by gRNA1 and gRNA2) or a region containing the variants (R2-del2 by gRNA3 and gRNA2). B. Taqman^™^ qRT-PCR analysis of gene expression in R2-del cells. C. Glucose stimulated insulin secretion (GSIS) assay. Data are mean ± SEM. *, *P* < 0.05; **, *P* < 0.01; ***, *P* < 0.005. Fold change of secreted insulin. Data were normalized to insulin secretion at basal level (0.5 mM glucose). *n* = 3.

Whilst the above results may reflect an action of the genomic deletions on chromatin accessibility and transcription, it is also conceivable that the attendant change in the structure of the ZnT8 protein, notably deletion of part of the C-terminus (encoded by exon 8), may affect protein dimerization and hence secretory granule zinc uptake. This, in turn, may have consequences for regulated insulin secretion [14, 15]. We therefore examined the impact of manipulating the neighbouring R2 region, which bears rs35859536. This region lies downstream of the *SLC30A8* gene and, as such, its deletion does not affect the primary sequence of ZnT8 (Figure S3A). Similar to R1, R2 showed reduced transcriptional activity in promoter-luciferase assays (Figure S5A–S5C). However, the DNA fragment (F4) bearing rs35859536 had higher transcriptional activity than other regions within the R2 region (Figure S5B and S5C). Deletion of R2 (Figure 4A and Figure S5D–S5G) from EndoC-βH3 cells also lowered *SLC30A8* and nearby gene transcript levels, as observed after R1-deletion (Figure 4B). R2 deletion also enhanced insulin secretion stimulated by glucose and IBMX (Figure 4C and 4D). Taken together, these data indicate that both R1 and R2 regulate the transcriptional activity of the enhancer cluster and impact the expression levels of *SLC30A8* and nearby genes.

### T2D-associated variants affect transcriptional activity

**Figure 5.**
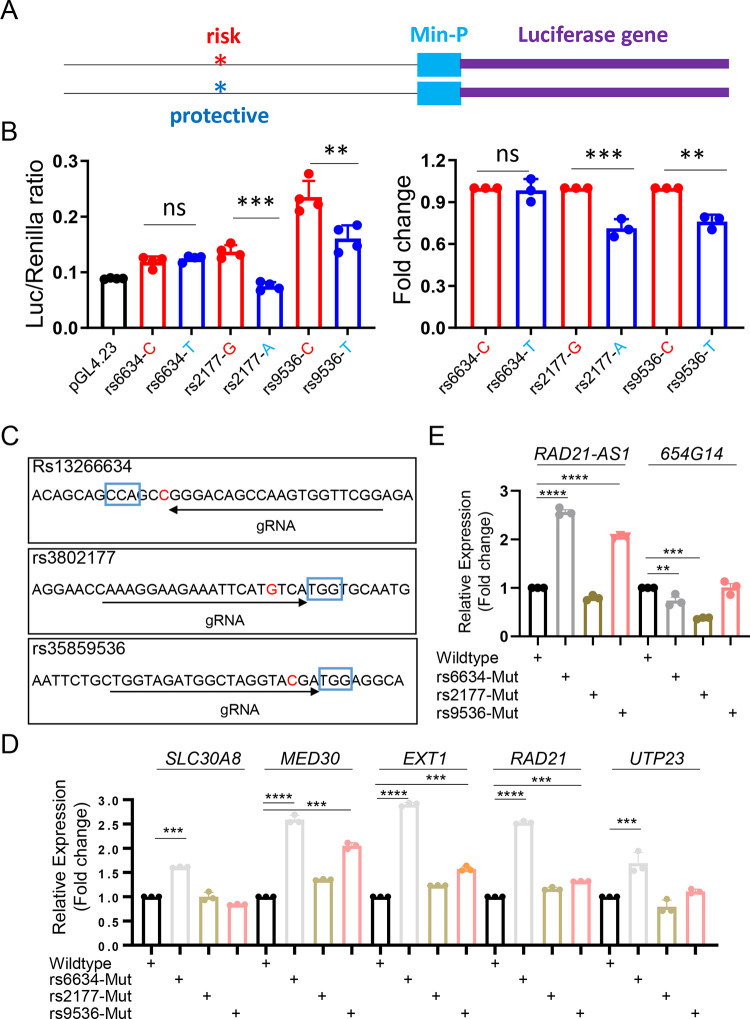
Function of individual variants in β-cells. A and B. Promoter-luciferase assay of individual variantss in EndoC-βH3 cells. A. Diagram of variant bearing genomic DNA fragment in pGL4.23 vector. B. promoter-luciferase assay of the risk or protective variant in EndoC-βH3 cells. C. Diagram of blunt mutation at the variants by CRISPR-Cas9 mediated genome editing in EndoC-βH3 cells. D. Taqman^™^ RT-qPCR analysis of *SLC30A8* and nearby genes. E. SYBR green qRT-PCR analysis of lncRNAs. Data are mean ± SEM. *, *P* < 0.05; **, *P* < 0.01; ***, *P* < 0.005. *n* = 3.

We next investigated the roles of the diabetes-associated variants in β cells. *In silico* analyses of transcription factor (TF) binding at the variant sites indicated that there may be differential TF binding (Table 4) which might affect the transcriptional activity of the active enhancers in which they reside. We first analysed the transcriptional activity of three variants in EndoC-βH3 cells (Figure 5A and 5B). The 200–300 bp DNA fragments surrounding either risk or protective variant were cloned into the vector pGL4.32 (Figure 5A) and the changes confirmed by Sanger sequencing (Figure S6A). Promoter-luciferase assays in EndoC-βH3 cells (Figure 5B) revealed that the protective variants of rs3802177 and rs35859536 displayed lower luciferase activity compared with their risk variants. No change (risk vs protective variant) was detected for rs13266634.

To determine whether these variants influenced the expression of *SLC30A8* and / or nearby genes, we sought to introduce these variants into a β-cell line. However, both EndoC-βH1 and EndoC-βH3 cells are homozygous for the risk forms of the three variants and the introduction of the protective variants as “point mutations” by CRISPR-Cas9 engineering was not feasible. To assess whether these risk variants may nevertheless have the potential to influence transcriptional activity, we created disruptive mutations at each variant site, with high efficiency, through conventional CRISPR-Cas9 genome editing (Figure 5C, S6B and S6C). Introduction of the mutations altered the levels of transcripts at the *SLC30A8* locus as examined by TaqMan qPCR or SYBR-green qPCR (Figure 5D and 5E). Notably, disruption of rs13266634 led to higher expression not only of *SLC30A8* but also of other transcripts. Disruption of rs35839536 also caused significant changes at several genes, whereas rs3802177 disruption was without effect, though this may possibly be due to the lower efficiency of CRISPR-Cas9 genome editing at this site (Figure S6C).

### Genes within the TAD domain are required for cell survival

**Figure 6.**
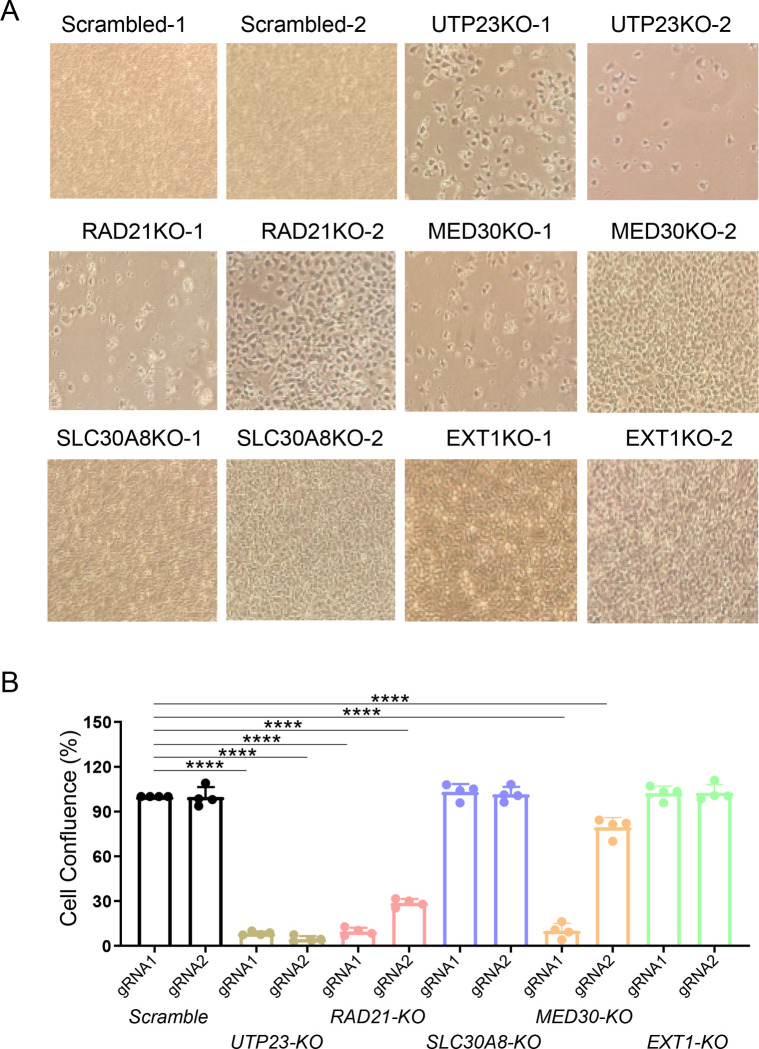
Impact of gene inactivation on cell survival. Two gRNAs were designed for each gene, cloned individually into pLenti-RIP-Cas9-BSD vector and delivered into 1 million EndoC-βH3 cells via lentiviral approach. Cells were selected with Blasticidin (25 μg/ml final concentration) for a week and cultured continuously for another 3 weeks. A. Morphologies of gene knockout cells. Photos were taken at the end of 4-week culture. Note that cells with scrambled gRNAs were confluent, along with *SLC30A8* and *EXT1 KO* cells while *RAD21*, *MED30* and *UTP23* had significantly fewer surviving cells. B. Statistical analysis of surviving cells. 4 small areas on each plate were randomly selected and survival cells were counted and compared with the Scrambled samples. Data are mean ± SEM. *, *P* < 0.05; **, *P* < 0.01; ***, *P* < 0.005; ****, *P* < 0.001.

Based on chromatin accessibility and the presence of regulatory marks in human islets (Figure 2A and S3A), the *SLC30A8* super-enhancer cluster is likely to co-regulate the *SLC30A8* gene as well as several nearby genes. Of these, the role of only *RAD21* [32] has previously been explored in β cells. We therefore inactivated each gene individually in EndoC-βH3 cells using CRISPR-Cas9 (Figure 6A, 6B and Figure S7A–S7C). Inactivation of *RAD21* and *UTP23* resulted in a significant reduction of cell viability when compared with scrambled gRNA-treated control cells (Figure 6A and 6B). *MED30* inactivation had a similar impact, although the effects on cell viability observed using gRNA2 were less marked than with the other guide RNAs deployed (Figure 6A and 6B). In comparison, cells null for *SLC30A8* or *EXT1* showed no apparent defects in survival or growth (Figure 6A, 6B and Figure S8A and S8B).

## Discussion

### GWAS-identified genetic variants affect the expression of the SLC30A8 gene

In this study, we first assessed the effects of T2D GWAS variants on *SLC30A8* gene expression in human islet samples. Previous reports [15, 18, 19, 38] have focused almost exclusively on the lead variant rs13266634 which alters the amino acid sequence of ZnT8 (R325W), or on rare loss-of function *SLC30A8* variants [22, 39]. Whilst previous eQTL analyses [27, 40, 41] have failed to identify eQTLs for *SLC30A8*, analyses based on cASE identified significant allelic expression imbalance in *SLC30A8* [27]. Building on these earlier analyses [27], we confirm here that *SLC30A8* expression in human islets exhibits imbalanced allelic expression, where the T2D risk allele is more strongly expressed than the protective allele (Figure 1B). Among four reporter variants found in the *SLC30A8* transcript, rs11558471 displayed the most significantly imbalanced expression (*p*=4.64 × 10^−14^).

In contrast, we were unable to identify cASE of nearby genes when explored in the same collection of human islet samples [27]. Unlike *SLC30A8,* whose expression is restricted to the islet endocrine compartment [15], all neighbouring genes are expressed ubiquitously across tissues, including those co-isolated with human islets (ductal, acinar, mesenchymal etc. cells). Since the latter cell types comprise as much as 65 % of typical human islet preparations [41] our study may be underpowered to detect cASE in the genes neighbouring *SLC30A8*.

### The enhancer super-enhancer cluster may regulate multiple genes

Disease-associated variants are often enriched in tissue-specific enhancer clusters in disease-relevant cell types [42]. These regions assume a complex chromatin structure through DNA looping, regulating the expression of multiple genes. Assessed by HiC [30] and promoter-HiC [5] (Figure 2A and S3A), the enhancer cluster at the *SLC30A8* locus forms a unique 3D structure that has close contacts with multiple genes at both 5’ and 3’ ends. Although we did not detect significant associations between variants and nearby gene expression in cASE or eQTL analysis (see above), we found that forced changes in enhancer structure imposed by genomic editing of EndoC-βH3 cells affected the expression of nearby genes.

We focused on two variant-bearing regulatory regions (R1 and R2 enhancer elements) given that these are likely to exert the greatest effects on *SLC30A8* an nearby gene expression. These two regions were bound by β-cell specific transcription factors such as FOXA2 and NKX2.2 (for R1) and PDX1 (for R2) (Figure S3B) (http://pasqualilab.upf.edu/app/isletregulome) [28]. Although neither region displayed transcriptional activity *per se* (Figure S4 and S5), deletion of R1 and R2 led to reduced expression of *SLC30A8* and other nearby genes examined (Figure 3B and 4B). These data demonstrate that these regions affect the transcriptional activity of the enhancer cluster and in turn impact the expression of multiple genes. It is possible that these regions play other role(s) than classical transcriptional activity within the enhancer cluster. Indeed, the wider variant region including R1 and R2 is spatially associated with the CTCF-binding sites (“CBS”) at both the 5’ and 3’ ends of the enhancer cluster (Figure 2B). Thus, they may be involved in maintaining the chromatin structure of the enhancer. We have previously reported such a role for variants in the *STARD10* locus [13]. Although neither region at the *SLC30A8* locus displayed transcriptional activity *per se* (Figure S4B, S4C, S5B and S5C), each was bound by β-cell specific transcription factors such as FOXA2 and NKX2.2 (for R1) and PDX1 (for R2) (Figure S3B) (http://pasqualilab.upf.edu/app/isletregulome) [28]. Correspondingly, deletion of R1 and R2 led to reduced expression of all genes examined (Figure 3B and 4B) suggesting that these two regions are critical for the transcriptional activity of the super-enhancer cluster.

### Enhancer-bearing variants affect transcriptional activity and alter the expression of SLC30A8 and other genes

In this study, we focused on three enhancer-bearing variants and assessed their functions in β cells. Variants within the enhancer cluster are expected to alter transcriptional activity and, in turn, influence the levels of downstream transcript(s). The T2D protective alleles of rs3802177 and rs35859536 exhibited lower transcriptional activities in promoter-luciferase assays (Figure 5A and 5B) which is consistent with the cASE results. Furthermore, disruption of two variants, rs13266634 and rs35859538, led to significant changes on the expression levels of *SLC30A8* and nearby genes. These data suggest that these variants could affect the overall transcriptional activity of the enhancer cluster. As listed in Table 4, the variant sites are likely bound by different transcription factor(s) between risk and protective variants, potentially changing the nearby transcriptional landscape. We note that, of these variants, rs13266634 hosts the majority of the posterior probability in the credible set (Table 3) [3], and as such it is unclear to what extent the neighbouring variants – and their independent effects on gene expression – contribute to disease risk. In any case, future work will be needed to determine whether differential transcription factor binding in this region impacts local chromatin structure and enhancer activity.

### Genes regulated by the super-enhancer cluster are critical for β-cell survival

Previous studies [27], and the present work, are consistent with the view that variants in *SLC30A8* influence transcriptional activity. The observation that protective variants are associated with lower *SLC30A8* mRNA levels suggests that the variants in the associated enhancer could also influence the transcriptional activity of nearby genes. These include *RAD21*, *MED30* and *UTP23*, which are involved in chromatin organization [32], transcription [34] and translation [33], respectively. Correspondingly, we show that inactivation of the latter genes exerts deleterious effects on β cell survival. However, the effects of smaller or more transient decreases in their expression, which might conceivably exert positive effects on cell survival or function, were not explored.

Inactivation of *SLC30A8* had no evident impact on β-cell survival under the conditions used here, suggesting that the effects of altered *SLC30A8* levels are due to more acute effects on insulin secretion. Nevertheless, we note that, in a recent study [23], inactivation of *SLC30A8* in human stem cell-derived β-like cells (or the introduction of the loss-of-function R138X mutation) was protective against apoptosis under conditions of limiting Zn^2+^. Likewise, ZnT8 inactivation in mice protected islets from hypoxia and treatment with cytotoxic cytokines [43]. These earlier reports indicate that *SLC30A8* may influence cell viability under the stressed conditions which pertain in diabetes.

### Conclusions

We examined the role in pancreatic β cells of a previously-identified islet super-enhancer cluster at the *SLC30A8* locus. We demonstrate that this cluster regulates the expression of *SLC30A8* and, at least *in vitro*, that of nearby genes involved in fundamental cellular processes. Future studies will be needed to confirm or refute a possible role for the latter in the effects exerted by T2D-associated variants.

## Materials and Methods

### Expression quantitative trait loci (eQTL) analysis

Expression quantitative trait *loci* analyses were performed using two previously-published datasets [40, 44]. For Khamis et al., 2019, a total of 100 organ donors and 103 living donors of European descent were included in the study, with genotyping data (2.5M Omniarray Beadchip) from blood. We utilised the Genome-studio software to call the genotypes and QC thresholds for SNPs were as follows: a Hardy-Weinberg equilibrium > 0.001, a minor allele frequency > 0.05 and a call rate > 0.95. Imputation was performed using Impute2 (v2.3.2) from the 1,000 genomes panel (phase 3). Following QC, > 8.7M SNPs remained for further analysis. Ethnicity was tested using principal component analysis from 1,000 genomes and confirmed the European descent of subjects, therefore, population structure was not adjusted for. For the organ donors, RNA was isolated using collagenase, and in living donors isolated using laser capture microdissection (LCM). RNA gene expression was determined using Affymetrix (Human Genome U133 Plus 2.0 Array). RNA expression data was normalised using the Robust Multichip Analysis (RMA) method utilising the package affy (PMID: 14960456) and transcriptomic batch effect was corrected for using the *combat* approach using the sva R package (Leek et al., 2012: PMID: 22257669). Genotyping and RNA data was integrated using the fastQTL software to identify eQTLs, adjusted for sex and age. P-values were computed using a permutation pass, with the number set from 1,000 to 10,000. Multiple testing was accounted for using Benjamini-Hochberg method to correct p-values. We report the nominal p-values for the tested eQTLs. For Vinuela et al [40], we utilised data published from a total of 26 preparations of FAC-sorted pancreatic beta cells and genotyped using the 2.5M Omniarray Beadchip. The eQTL analysis was performed using fastQTL, with a cis-window of 1 Mb, and adjusting for sex, batch effect (for RNA sequencing), PCs (for genotyping) and laboratory origin of samples. P-values were determined based on the 1,000 permutations per gene.

### Combined allele-specific expression (cASE) of transcript

The analysis of combined allele-specific expression (cASE) in multiple samples was performed as described in [27]. In brief, in all samples that are heterozygous for genomic variants within transcribed regions (reporter variants), the number of RNA-seq reads containing either the reference or alternative alleles is quantified. Then, their binomial significances are used to calculate a weighted Z-score, which measures the significance of the allelic bias across all samples, using the RNA-seq read coverage of each sample as weight. Significance is assessed by comparing the obtained Z-score with a control distribution created using the same read counts, but randomly shuffled across heterozygous individuals 1000 times.

All genomic variants located in the same Topologically Associated Domain (TAD) of each reporter variant are assessed as candidate variants responsible for the cASE effect. To do so, all samples that have a heterozygous reporter are separated in two subgroups, based on whether they are heterozygous or homozygous for the candidate variant. The Z-score is then calculated for each subgroup, using the heterozygous reporter variant. If the Z-score of the heterozygous candidate subgroup is more significant than the reporter’s Z-score (which was calculated with all the samples), and if the Z-score of the homozygous candidate subgroup is non-significant, the variant is then proposed as a putative candidate for mediating the combined allele-specific expression effect.

### Cell culture

The human-derived β cell line EndoC-βH3 was grown on extracellular matrix (ECM; 1% v/v) and fibronectin (2 μg/ml)-coated plates or petri dishes in serum-free DMEM containing low glucose (1 g/L), 2% (w/v) albumin from bovine serum fraction V, 50 μM β-Mercaptoethanol, 10 mM nicotinamide, 5.5 μg/mL human transferrin, 6.7 ng/mL sodium selenite, penicillin (100 units/mL), and streptomycin (100 μg/mL) [45]. HEK293T cell was cultured in DMEM high glucose (4500 mg/L) medium supplemented with 10% fetal bovine serum, 6 mM L-glutamine, penicillin (100 μg/mL) and streptomycin (100 μg/mL).

### CRISPR-Cas9-mediated genome editing

gRNA sequences were designed using the software provided by Broad Institute (https://portals.broadinstitute.org/gpp/public/analysis-tools/sgrna-design) [46]. To generate mutations in EndoC-βH3 cells, lentiviral constructs carrying a gRNA and humanized *S. pyogenes* Cas9 (*hsp*Cas9) were transfected into HEK293T cells together with packaging plasmids pMD2.G and psPAX2 using CaCl_2_ transfection protocol [47]. Scramble gRNAs were served as a SHAM control (Table S2). Next day, cells were treated with sodium butyrate (10 mM) for 8 hours before changing to fresh medium. The medium was collected twice in the next three days and subjected to ultracentrifugation (Optima XPN-100 Ultracentrifuge, Beckman Coulter) at 26,000 rpm for 2 hours at 4°C. The lentiviruses were collected from the bottom of the tube and titrated. Same number of viruses was used to transduce to the EndoC-βH3 cells (MOI = 6–8). Blasticidin (25 μg/ml) was added 72 h after infection to select lentivirus-infected cells.

For deletion of genomic regions, two lentiviral vectors were used. The first one (plenti-RIP-Cas9-BSD) carried a gRNA, Cas9 and Blasticidin resistant genes) and anther vector carried second gRNA with Hygromycin resistant gene and GFP gene cassette). Both plasmids were co-transfected into HEK293T cells with packaging plasmids. After infection, cells were monitored by GFP positivity and selected by both Blasticidin and hygromycin at final concentration of 25 μg/ml and 200 μg/ml, respectively. All gRNA sequences in this study are listed in Table S2.

### PCR and qRT-PCR

Fusion high fidelity Taq polymerase (Thermo Fisher Scientific) was used in all routine PCR reactions to avoid PCR errors. A typical PCR reaction was set as follow: 98°C for 30 s, then with 35 cycles at 98°C 10 s, 60°C 10 s and 72°C 15 s. The primer sets for genomic DNA amplification are listed in Table S3.

Total RNA from EndoC-βH3 cells was obtained using TRIzol reagent (Invitrogen). Total RNAs (2 μg) were then reverse-transcribed into first strand cDNAs using High-Capacity cDNA Reverse Transcription Kit (Thermo Fisher Scientific) according to the manufacturer’s instructions. Real-time PCR was performed on a 7500 Fast Real-Time PCR System using the Fast SYBR^™^ Green master mix or Fast Taqman^™^ master mix. The Taqman gene expression primer/probe sets (Thermos Fisher) are listed in Table S1. The SYBR^™^ Green PCR primer sets are listed in Table S4. The experiment was performed in duplicate and repeated three times.

### Molecular cloning

Regulatory regions (R1 and R2), identified by integration of previously published human islet ATAC-seq and H3K27ac ChIP-seq datasets [5], were PCR-amplified from genomic DNA extracted from EndoC-βH3 cells with primer sets (Table S5) designed by Primer3-based software and cloned into pGL4.23[*luc*/minP] vector (Promega) between KpnI and XhoI restriction enzyme sites. Plasmid DNA was extracted using mini-prep plasmid extraction kit and/or Maxi-prep plasmid extraction kit (QIAGEN). The constructs were further confirmed by the Sanger sequencing.

### Transfection and luciferase assay

EndoC-βH3 cells were seeded at a density of 50,000 per well in 96-well plates. After 48 hours, 0.1 μg of luciferase constructs containing putative regulatory sequences were co-transfected with 1 ng of pRL-Renilla construct as internal control into EndoC-βH3 cells, using Lipofectamine 2000, according to manufacturer’s instruction. pGL4.23 empty vector was served as a control. 48 h later, transfected cells were washed once with PBS and lysed directly in passive cell lysis buffer (Promega). Cells were incubated on a rotating platform at room temperature for 10 min. to ensure complete lysis of cells, and then spun at 10,000 rpm for 10 min to remove cell debris. Supernatant was transferred into a fresh tube and used to measure luciferase activity with Dual-Luciferase Reporter Assay kit (Promega) on a Lumat LB9507 luminometer (Berthold Technologies). Firefly luciferase measurements were normalized to *Renilla* luciferase.

### Insulin secretion

EndoC-βH3 cells were seeded onto ECM/Fibronectin-coated 96-well plates at 7.0 × 10^4^ cells per well. Two days after seeding, cells were incubated overnight in a glucose starving medium (glucose-free DMEM supplemented with 2% Albumin from bovine serum fraction V, 50 μl 2-mercaptoethanol, 10 mM nicotinamide, 5.5 μg/ml transferrin, 6.7 ng/ml sodium selenite, 100 units/ml, penicillin, 100 μg/ml streptomycin and 2.8 mM glucose). The next morning cells were incubated for 1 h in Krebs-Ringer solution [0.2% BSA, 25% solution 1 (460 mM NaCl), 25% solution II (96 mM NaHCO_3_, 20 mM KCl and 4 mM MgCl_2_), 25% solution III (4 mM CaCl_2_), 10 mM HEPES] supplemented with 0.5 mM glucose. EndoC-βH3 cells were then incubated in the presence of low (0.5 mM) or high glucose (15 mM) with or without 0.5 mM IBMX. After incubation for 1 h, the supernatant was collected, placed onto ice and centrifuged for 5 min. at 3,000 rpm at 4°C. The supernatant was then transferred into a fresh tube. Cells were lysed in 50 μL cell lysis solution (TETG: 20 mM Tris pH 8.0, 1% Triton X-100, 10% glycerol, 137 mM NaCl, 2 mM EGTA). The lysate was then removed to a fresh tube and centrifuged at 3,000 rpm for 5 min at 4°C. Insulin content was measured using an insulin ultra-sensitive assay kit (Cisbio). Secreted insulin was normalized as percentage of total insulin content. Fold increase in glucose- or other stimuli-stimulated insulin secretion is expressed as a ratio in comparison with secretion at basal level (0.5 mM glucose). Insulin secretion assays were performed in duplicate with insulin measurement in duplicate as well.

### Transcription factor binding motif analysis

TF binding profile on genetic variants was carried out using JASPAR CORE program (http://jaspar.genereg.net) [37, 48]. The threshold of relative profile score was set up at 80%. The scores of potential transcription factors were compared between risk and protective variants and listed in Table 4.

### Quantification and statistical analysis

Data are expressed as means ± SEM. Significance was tested by Student’s two-tailed t test (paired or none-paired, Mann-Whitney test for non-parametric data, and one- or two-way ANOVA with SIDAK multiple comparison test, as appropriate, using Graphpad Prism 9.0 software. p < 0.05 was considered significant. The statistical details can be found in the figure legends (indicated as *n* number).

## Supplementary Material

Supplement 1

Supplement 2

## Figures and Tables

**Table 1A:** eQTL analysis data set from laser capture cohort [44]

rsID	REF	ALT	Distance (Kb)	Nominal P	FDR	beta	Gene Symbol
rs3802177	G	A	326852	6.37 × 10^−3^	0.442	0.25	RAD21
rs13266634	C	T	298121	6.37 × 10^−3^	0.442	0.18	MIR3610 / RAD21-AS1
rs4300038	G	A	359742	6.66 × 10^−3^	0.449	0.26	RAD21
rs13266634	C	T	37216	7.88 × 10^−3^	0.476	0.36	SLC30A8
rs9650069	C	T	345847	8.36 × 10^−3^	0.485	0.25	RAD21
rs11558471	A	G	299071	8.93 × 10^−3^	0.495	0.16	MIR3610 / RAD21-AS1
rs3802177	G	A	37458	1.31 × 10^−2^	0.555	0.33	SLC30A8
rs13266634	C	T	326610	1.45 × 10^−2^	0.570	0.22	RAD21
rs35859536	C	T	333302	1.55 × 10^−2^ 0.0154994	0.585	0.22	RAD21
rs11558471	A	G	327560	1.58 × 10^−2^	0.583	0.21	RAD21
rs11558471	A	G	406989	1.66 × 10^−2^	0.591	0.11	UTP23
rs3802177	G	A	298363	1.7 × 10^−2^	0.602	0.16	MIR3610 / RAD21-AS1
rs13266634	C	T	400600	1.84 × 10^−2^	0.606	0.18	UTP23
rs35859536	C	T	43908	2.36 × 10^−2^	0.641	0.31	SLC30A8
rs9650069	C	T	317358	2.47 × 10^−2^	0.648	0.15	MIR3610/ RAD21-AS1
rs35859536	C	T	304813	2.54 × 10^−2^	0.652	0.15	MIR3610 / RAD21-AS1
rs4300038	G	A	331253	3.68 × 10^−2^	0.703	0.14	MIR3610 / RAD21-AS1
rs13266634	C	T	326609	4.02 × 10^−2^	0.715	0.077	RAD21
rs11558471	A	G	38166	4.13 × 10^−2^	0.718	0.26	SLC30A8
rs3802177	G	A	400842	4.15 × 10^−2^	0.719	0.16	UTP23
rs16889471	G	A	303958	4.53 × 10^−2^	0.730	−0.15	MIR3610 / RAD21-AS1
rs13266634	C	T	−3724	4.90 × 10^−2^	0.734	0.15	SLC30A8

**Table 1B:** eQTL analysis of organ donors (beta cells) [40]

rsID	REF	ALT	Distance (Kb)	Nominal P value	FDR	beta	Gene Symbol
rs16889471	G	A	−933472	5.49 × 10^−3^	0.927	−0.68	EXT1
rs11774700	T	C	441106	1.74 × 10^−2^	0.942	−0.57	EIF3H
rs11558471	A	G	298694	2.06 × 10^−2^	0.945	−0.83	MIR3610
rs13266634	C	T	−939309	2.52 × 10^−2^	0.949	0.61	EXT1
rs3802177	G	A	−939067	2.52 × 10^−2^	0.949	0.61	EXT1
rs4300038	G	A	−906177	2.52 × 10^−2^	0.949	0.61	EXT1
rs9650069	C	T	−920072	2.52 × 10^−2^	0.949	0.61	EXT1
rs11558471	A	G	406569	2.59 × 10^−2^	0.945	−0.50	EIF3H
rs13266634	C	T	405619	2.66 × 10^−2^	0.950	−0.50	EIF3H
rs3802177	G	A	405861	2.66 × 10^−2^	0.950	−0.50	EIF3H
rs4300038	G	A	438751	2.66 × 10^−2^	0.950	−0.50	EIF3H
rs9650069	C	T	424856	2.66 × 10^−2^	0.950	−0.50	EIF3H
rs35859536	C	T	15268	2.71 × 10^−2^	0.951	0.29	RN7SL826P
rs35859536	C	T	−932617	2.90 × 10^−2^	0.950	0.63	EXT1
rs13266634	C	T	−348169	3.09 × 10^−2^	0.952	0.67	MED30
rs3802177	G	A	−347927	3.09 × 10^−2^	0.953	0.67	MED30
rs4300038	G	A	−315037	3.09 × 10^−2^	0.953	0.67	MED30
rs9650069	C	T	−328932	3.09 × 10^−2^	0.953	0.67	MED30
rs11558471	A	G	−347219	3.27 × 10^−2^	0.954	0.67	MED30
rs11774700	T	C	441106	3.57 × 10^−2^	0.955	−0.39	EIF3H
rs13266634	C	T	8576	4.88 × 10^−2^	0.960	0.25	RN7SL826P
rs3802177	G	A	8818	4.88 × 10^−2^	0.960	0.25	RN7SL826P
rs4300038	G	A	41708	4.88 × 10^−2^	0.960	0.25	RN7SL826P
rs9650069	C	T	27813	4.88 × 10^−2^	0.960	0.25	RN7SL826P
rs11774700	T	C	−903822	4.98 × 10^−2^	0.960	0.46	EXT1
rs11774700	T	C	−903822	4.98 × 10^−2^	0.960	−0.51	EXT1

**Table 1C:** eQTL analysis from TIGER cohort [27]

rsID	REF	ALT	Gene	Nominal P	FDR	Effect (Z)
rs13266634	C	T	SLC30A8	0.367	NS	[C] 0.903
rs13266634	C	T	MED30	0.979	NS	[C] 0.026
rs13266634	C	T	EXT 1	0.952	NS	[C] 0.06
rs13266634	C	T	RAD21-AS1	0.784	NS	[C] 0.274
rs3802177	G	A	EXT 1	0.938	NS	[G] 0.078
rs3802177	G	A	RAD21-AS1	0.873	NS	[G] 0.159
rs35859536	C	T	EXT 1	0.873	NS	[C] −0.055
rs35859536	C	T	RAD21-AS1	0.961	NS	[C] −0.05
rs11558471	A	G	SLC30A8	0.594	NS	[G] −0.534
rs11558471	A	G	MED30	0.905	NS	[G] 0.119
rs11558471	A	G	MiR3610	NA	NA	NA
rs11558471	A	G	RAD21-AS1	0.886	NS	[G] −0.144
rs11558471	A	G	RP11-654G14.1	0.648	NS	[G] 0.457
rs4300038	G	A	MED30	0.892	NS	[G] −0.136
rs4300038	G	A	EXT 1	0.863	NS	[G] −0.173
rs4300038	G	A	RAD21-AS1	0.949	NS	[G] −0.065
rs9650069	C	T	MED30	0.817	NS	[C] −0.232
rs9650069	C	T	RAD21-AS1	0.770	NS	[C] −0.293
rs9650069	C	T	EXT 1	0.973	NS	[C] −0.034
rs11774700	T	C	MED30	0.562	NS	[C] −0.58
rs11774700	T	C	RAD21-AS1	0.791	NS	[C] 0.265
rs11774700	T	C	EXT 1	0.538	NS	[C] 0.616

**Table 1D:** eQTL analysis from InsPIRE cohort [49]

SNP	REF	ALT	Gene	P value	FDR	Effect (Z)
rs 13266634	C	T	SLC30A8	0.390	NA	[T] −0.016398
rs 13266634	C	T	MED30	0.038	NS	[T] −0.058180
rs 13266634	C	T	EXT1	0.603	NA	[T] 0.0124346
rs 13266634	C	T	RAD21-AS1	NA	NS	NA
rs3802177	G	A	EXT1	0.625	NA	[A] 0.0116709
rs3802177	G	A	RAD21-AS1	NA	NA	NA
rs35859536	C	T	EXT1	0.567	NA	[T] 0.0136128
rs35859536	C	T	RAD21-AS1	NA	NA	NA
rs 11558471	A	G	SLC30A8	0.361	NA	[G] −0.0172161
rs 11558471	A	G	MED30	0.024	NA	[G] −0.0624528
rs 11558471	A	G	MiR3610	NA	NA	NA
rs 11558471	A	G	RAD21-AS1	NA	NA	NA
rs 11558471	A	G	RP11-654G14.1	0.488	NA	[G] −0.025098
rs4300038	G	A	MED30	0.046	NA	[A] −0.055691
rs4300038	G	A	EXT1	0.592	NA	[A] 0.0127821
rs4300038	G	A	RAD21-AS1	NA	NA	NA
rs9650069	C	T	MED30	0.032	NA	[T] −0.059416
rs9650069	C	T	RAD21-AS1	NA	NA	NA
rs9650069	C	T	EXT1	0.695	NA	[T] 0.00933485
rs 11774700	T	C	MED30	0.025	NA	[C] −0.0641155
rs 11774700	T	C	RAD21-AS1	NA	NA	NA
rs 11774700	T	C	EXT1	0.276	NA	[C] 0.0266843

**Table 1E:** eQTL analysis from Atla et al [41]

rsID	REF	ALT	Gene	Nominal P	FDR	Effect (Z)
rs13266634	C	T	SLC30A8	0.268	NS	[T] −0.0394087
rs13266634	C	T	MED30	0.539	NS	[T] −0.0226174
rs13266634	C	T	EXT 1	NA	NA	NA
rs13266634	C	T	RAD21-AS1	NA	NA	NA
rs3802177	G	A	EXT 1	NA	NA	NA
rs3802177	G	A	RAD21-AS1	NA	NA	NA
rs35859536	C	T	EXT 1	NA	NA	NA
rs35859536	C	T	RAD21-AS1	NA	NA	NA
rs11558471	A	G	SLC30A8	0.211	NS	[G] −0.0439515
rs11558471	A	G	MED30	0.686	NS	[G] −0.0439515
rs11558471	A	G	MiR3610	NA	NA	NA
rs11558471	A	G	RAD21-AS1	NA	NA	NA
rs11558471	A	G	RP11-654G14.1	0.162	NS	[G] −0.0717920
rs4300038	G	A	MED30	0.562	NS	[G] −0.0717920
rs4300038	G	A	EXT 1	NA	NA	NA
rs4300038	G	A	RAD21-AS1	NA	NA	NA
rs9650069	C	T	MED30	0.662	NA	[T] −0.0162419
rs9650069	C	T	RAD21-AS1	NA	NA	NA
rs9650069	C	T	EXT 1	NA	NA	NA
rs11774700	T	C	MED30	NA	NA	NA
rs11774700	T	C	RAD21-AS1	NA	NA	NA
rs11774700	T	C	EXT 1	NA	NA	NA

**Table 2:** cASE analysis of heterozygous human islet samples [27]

rsID	REF	ALT	Gene	P value	Z-score	Tag SNP	LD r2
rs11558471	G	A	SLC30A8	4.64 × 10^−14^	[A] 19.30	NA	NA
rs13266634	C	T	SLC30A8	2.88 × 10^−6^	[C]11.75	rs11558471	0.96
rs17738231	G	C	SLC30A8	1.06 × 10^−4^	[G] 9.60	rs11558471	0.06
rs75043555	C	G	SLC30A8	1.13 × 10^−4^	[C] −9.56	rs11558471	0.05

**Table 3:** Variant credible set at the *SLC30A8* locus

rsID	REF	ALT	Lead	MEGA_P	Posterior Probability	In credset?
rs13266634	C	T	rs13266634	3.22 × 10^−115^	0.99830716	YES
rs3802177	G	A	rs13266634	7.67 × 10^−114^	0.001647658	NO
rs11558471	A	G	rs13266634	4.38 × 10^−111^	2.97 × 10^−6^	NO
rs35859536	C	T	rs13266634	5.07 × 10^−113^	4.22 × 10^−5^	NO

**Table 4:** Candidate transcription factor and miRNA binding at variant sites

rsID	Risk variant	Protective variant
rs13266634(C/T)	PAX9	HIC2, ASCL1, NEUROD1
rs3802177(G/A)	CREB1	GATA6, LIN54, POU5F1
rs35859536(C/T)	E2F6, FOXC1, HLTF, HMBOX1	No
rs3802177(G/A)	No MiRNA binding	miR-190-5p or 190b-5p
rs11558471 (A/G)	miR-1248	miR-3074-5P
