# Supplementary material for: Multiple genetic variants at the SLC30A8 locus affect local super-enhancer activity and influence pancreatic β-cell survival and function
Source: bioRxiv. 2023 Oct 11:2023.07.13.548906. Originally published 2023 Jul 13. Preprint. [Version 2] doi: 10.1101/2023.07.13.548906 (PMC10369998; doi:10.1101/2023.07.13.548906)
Supplement: Supplement 2 [file media-2.pdf]

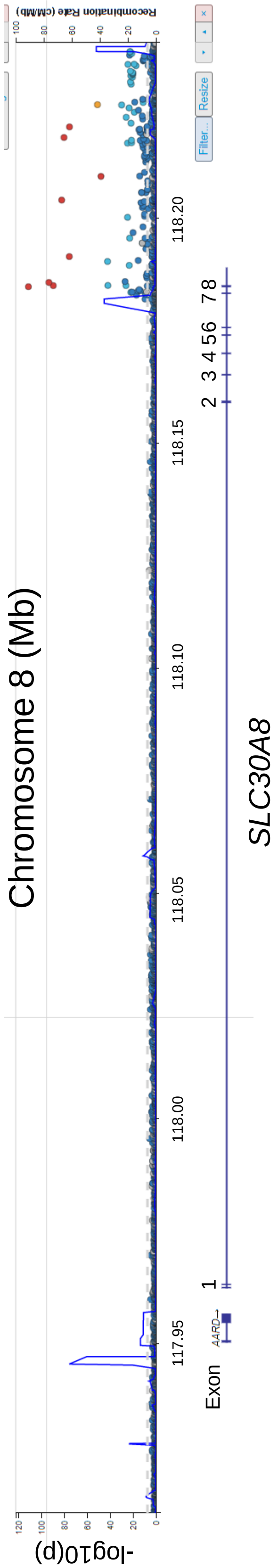

Chromosome 8 (Mb)
-log10(p)
Recombination Rate (cM/Mb)
117.95
118.00
118.05
118.10
118.15
118.20
Exon
AARD
1
2
3
4
56
78
SLC30A8
Filter...
Resize

Figure S1

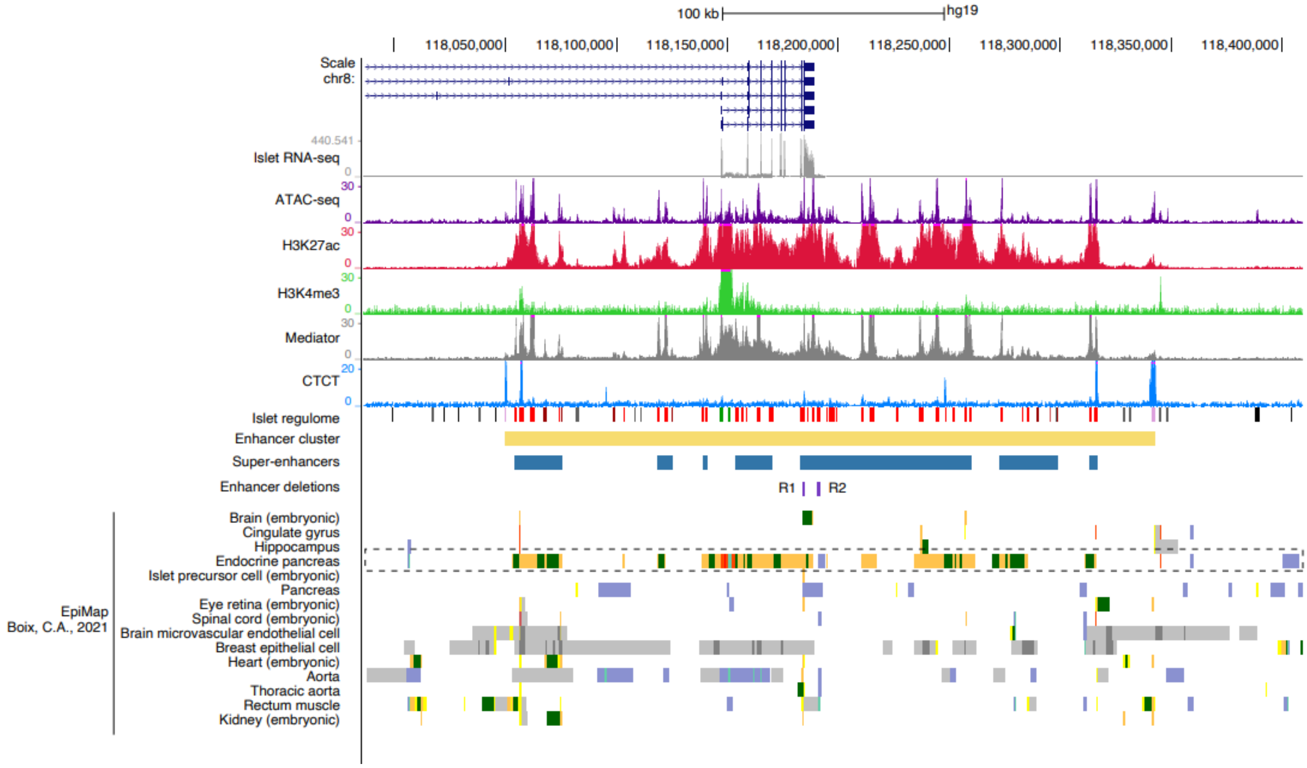
100 kb
hg19
118,050,000
118,100,000
118,150,000
118,200,000
118,250,000
118,300,000
118,350,000
118,400,000
Scale
chr8:
440.541
Islet RNA-seq
0
30
ATAC-seq
0
30
H3K27ac
0
30
H3K4me3
0
30
Mediator
0
20
CTCT
0
Islet regulome
Enhancer cluster
Super-enhancers
Enhancer deletions
R1
R2
EpiMap
Boix, C.A., 2021
Brain (embryonic)
Cingulate gyrus
Hippocampus
Endocrine pancreas
Islet precursor cell (embryonic)
Pancreas
Eye retina (embryonic)
Spinal cord (embryonic)
Brain microvascular endothelial cell
Breast epithelial cell
Heart (embryonic)
Aorta
Thoracic aorta
Rectum muscle
Kidney (embryonic)

Figure S2

A

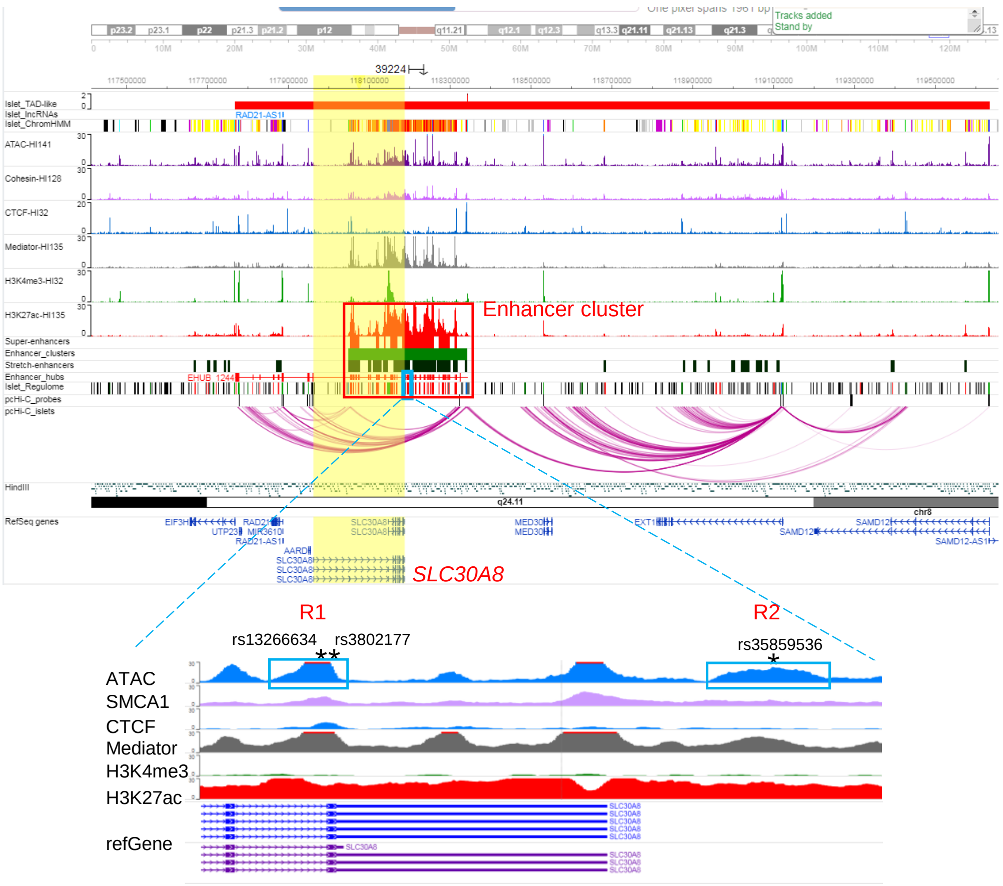

Enhancer cluster
SLC30A8
R1
R2
rs13266634
rs3802177
rs35859536
ATAC
SMCA1
CTCF
Mediator
H3K4me3
H3K27ac
refGene

B

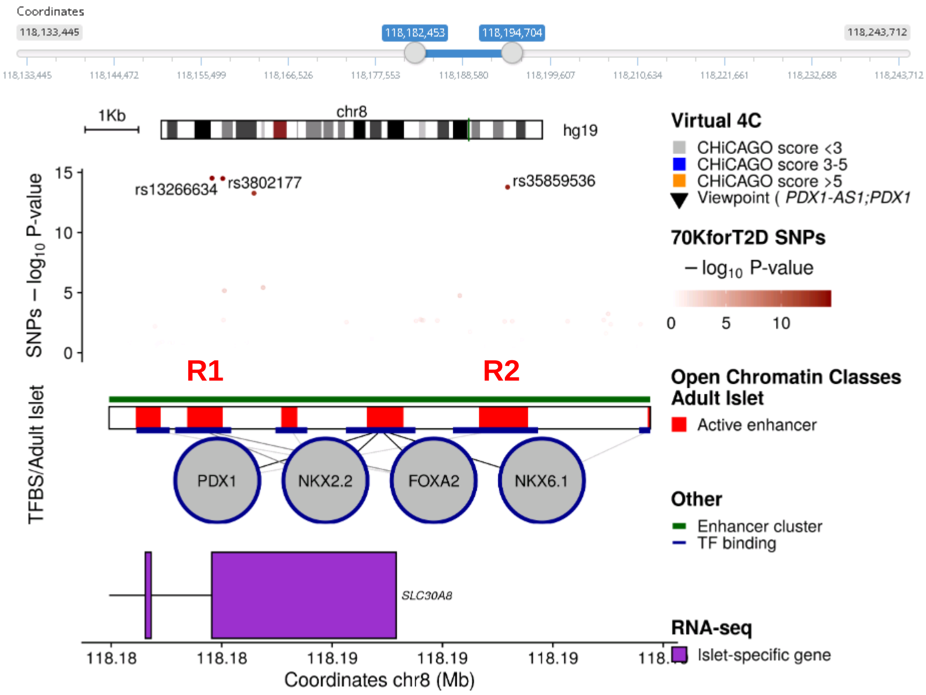

Coordinates
118,133,445
118,182,453
118,194,704
118,243,712
1Kb
chr8
hg19
Virtual 4C
CHiCAGO score <3
CHiCAGO score 3-5
CHiCAGO score >5
Viewpoint (PDX1-AS1;PDX1
70KforT2D SNPs
− log10 P-value
0
5
10
SNPs − log10 P-value
15
rs13266634
rs3802177
rs35859536
R1
R2
TFBS/Adult Islet
PDX1
NKX2.2
FOXA2
NKX6.1
Open Chromatin Classes Adult Islet
Active enhancer
Other
Enhancer cluster
TF binding
SLC30A8
RNA-seq
Islet-specific gene
118.18
118.19
Coordinates chr8 (Mb)

Figure S3

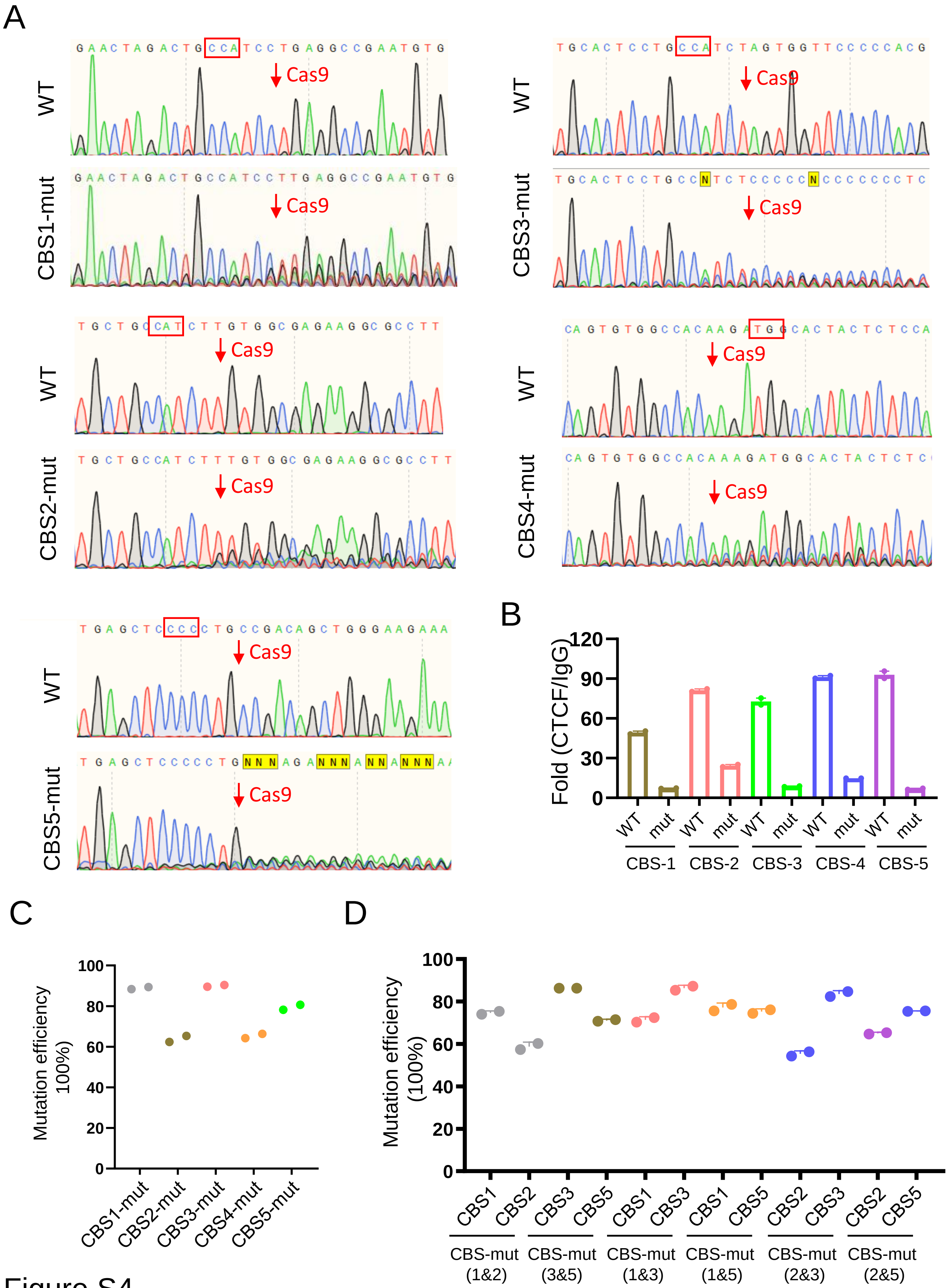
A
WT
CBS1-mut
CBS3-mut
CBS2-mut
CBS4-mut
CBS5-mut
Cas9
B
Fold (CTCF/IgG)
WT
mut
CBS-1
CBS-2
CBS-3
CBS-4
CBS-5
C
Mutation efficiency
100%)
CBS1-mut
CBS2-mut
CBS3-mut
CBS4-mut
CBS5-mut
D
Mutation efficiency
(100%)
CBS-mut (1&2)
CBS-mut (3&5)
CBS-mut (1&3)
CBS-mut (1&5)
CBS-mut (2&3)
CBS-mut (2&5)

Figure S4

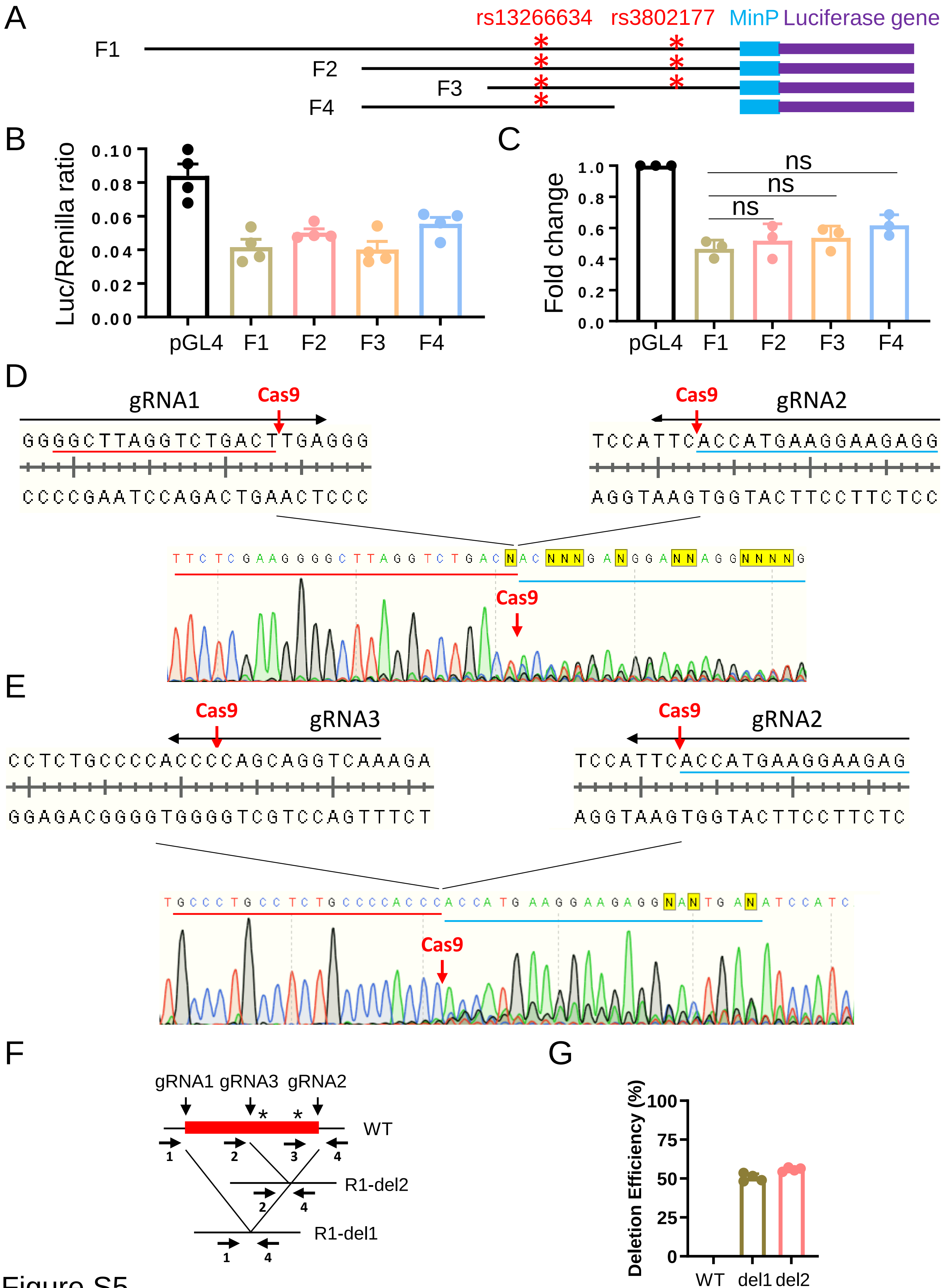
A
rs13266634
rs3802177
MinP
Luciferase gene
F1
F2
F3
F4
B
Luc/Renilla ratio
0.10
0.08
0.06
0.04
0.02
0.00
pGL4
F1
F2
F3
F4
C
Fold change
1.0
0.8
0.6
0.4
0.2
0.0
ns
ns
ns
pGL4
F1
F2
F3
F4
D
gRNA1
Cas9
GGGGCTTAGGTCTGACTTGAGGG
CCCCGAATCCAGACTGAACTCCC
Cas9
gRNA2
TCCATTCACCATGAAGGAAGAGG
AGGTAAGTGGTACTTCCTTCTCC
TTCTCGAAGGGGCTTAGGTCTGACNACNNNGANGGANNAGGNNNNG
Cas9
E
Cas9
gRNA3
CCTCTGCCCCACCCCAGCAGGTCAAAGA
GGAGACGGGGTGGGGTCGTCCAGTTTCT
Cas9
gRNA2
TCCATTCACCATGAAGGAAGAG
AGGTAAGTGGTACTTCCTTCTC
TGCCCTGCCTCTGCCCCACCCACCATGAAGGAAGAGGNANTGANATCCATC
Cas9
F
gRNA1
gRNA3
gRNA2
WT
1
2
3
4
R1-del2
2
4
R1-del1
1
4
G
Deletion Efficiency (%)
100
75
50
25
0
WT
del1
del2

Figure S5

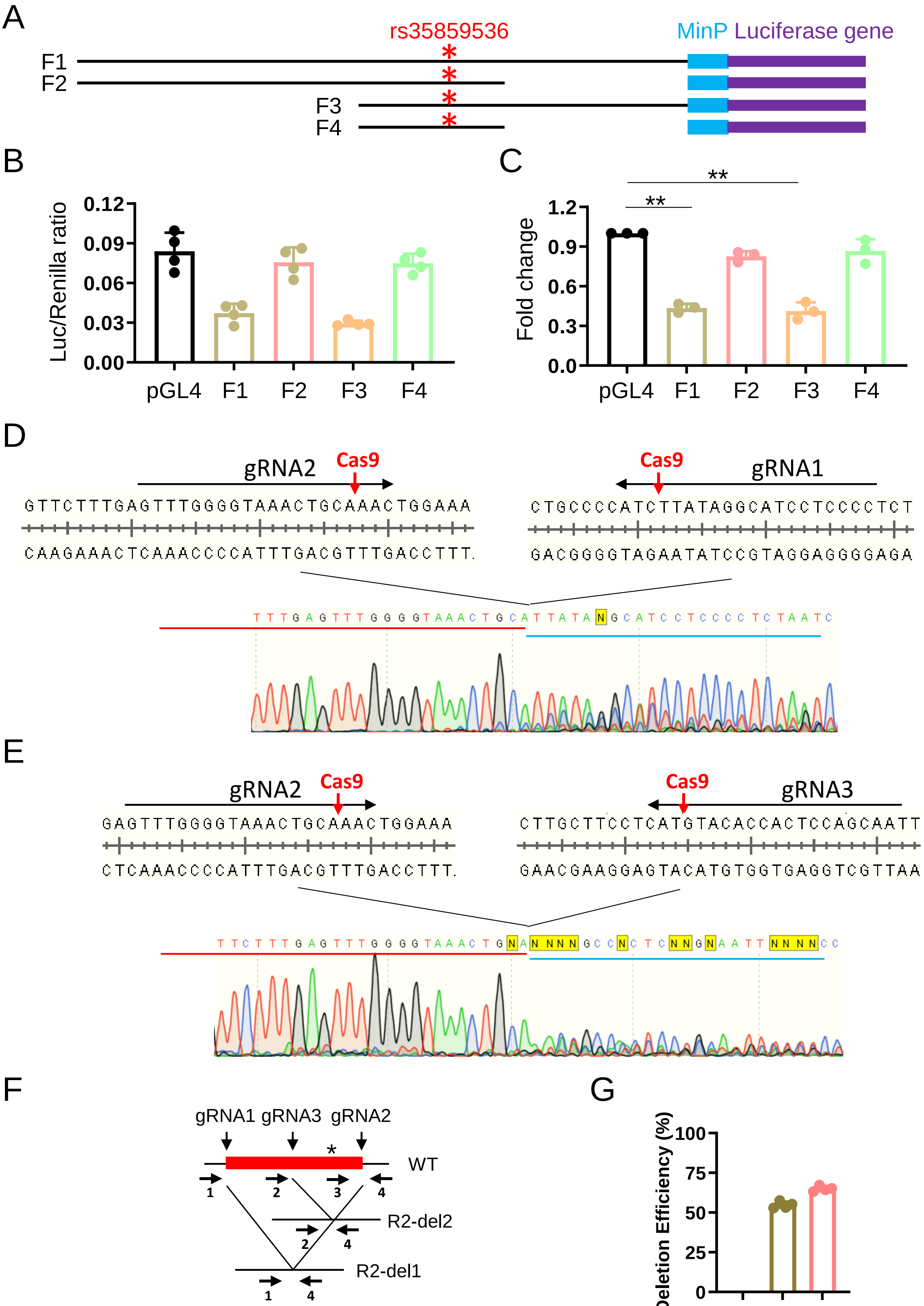
A
rs35859536
MinP Luciferase gene
F1
F2
F3
F4
B
Luc/Renilla ratio
0.12
0.09
0.06
0.03
0.00
pGL4 F1 F2 F3 F4
C
Fold change
1.2
0.9
0.6
0.3
0.0
**
**
pGL4 F1 F2 F3 F4
D
gRNA2
Cas9
Cas9
gRNA1
GTTCTTTGAGTTTGGGGTAAACTGCAAACTGGAAA
CAAGAAACTCAAACCCCATTTGACGTTTGACCTTT
CTGCCCCATCTTATAGGCATCCTCCCCTCT
GACGGGGTAGAATATCCGTAGGAGGGGAGA
TTTGAGTTTGGGGTAAACTGCATTATANGCATCCTCCCCTCTAATC
E
gRNA2
Cas9
Cas9
gRNA3
GAGTTTGGGGTAAACTGCAAACTGGAAA
CTCAAACCCCATTTGACGTTTGACCTTT
CTTGCTTCCTCATGTACACCACTCCAGCAATT
GAACGAAGGAGTACATGTGGTGAGGTCGTTAA
F
gRNA1 gRNA3 gRNA2
WT
1 2 3 4
R2-del2
2 4
R2-del1
1 4
G
Deletion Efficiency (%)
100
75
50
25
0
WT del1 del2

Figure S6

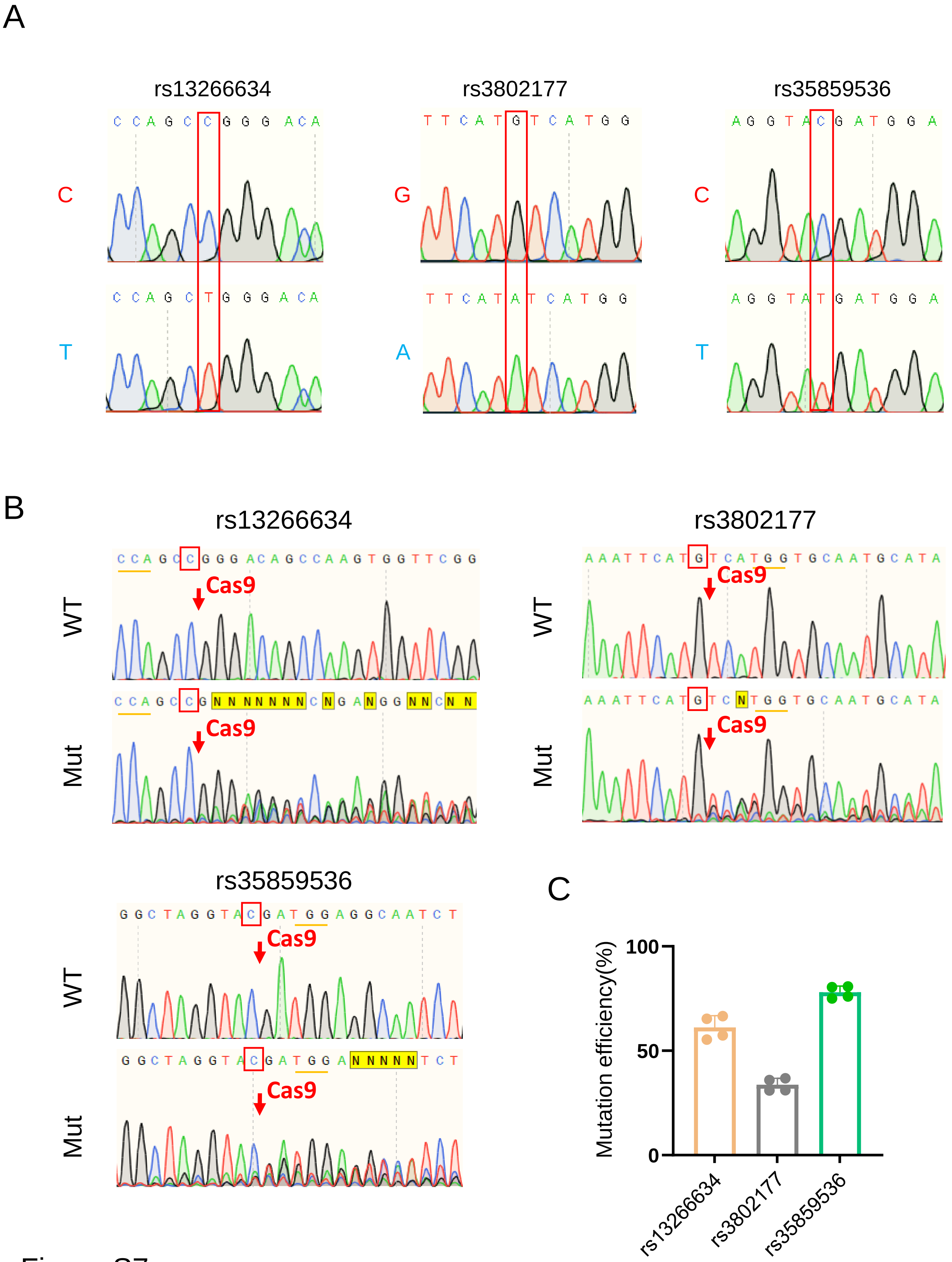
A
rs13266634
rs3802177
rs35859536
C
T
G
A
C
T
B
rs13266634
rs3802177
rs35859536
WT
Mut
Cas9
C
Mutation efficiency(%)
100
50
0
rs13266634
rs3802177
rs35859536

Figure S7

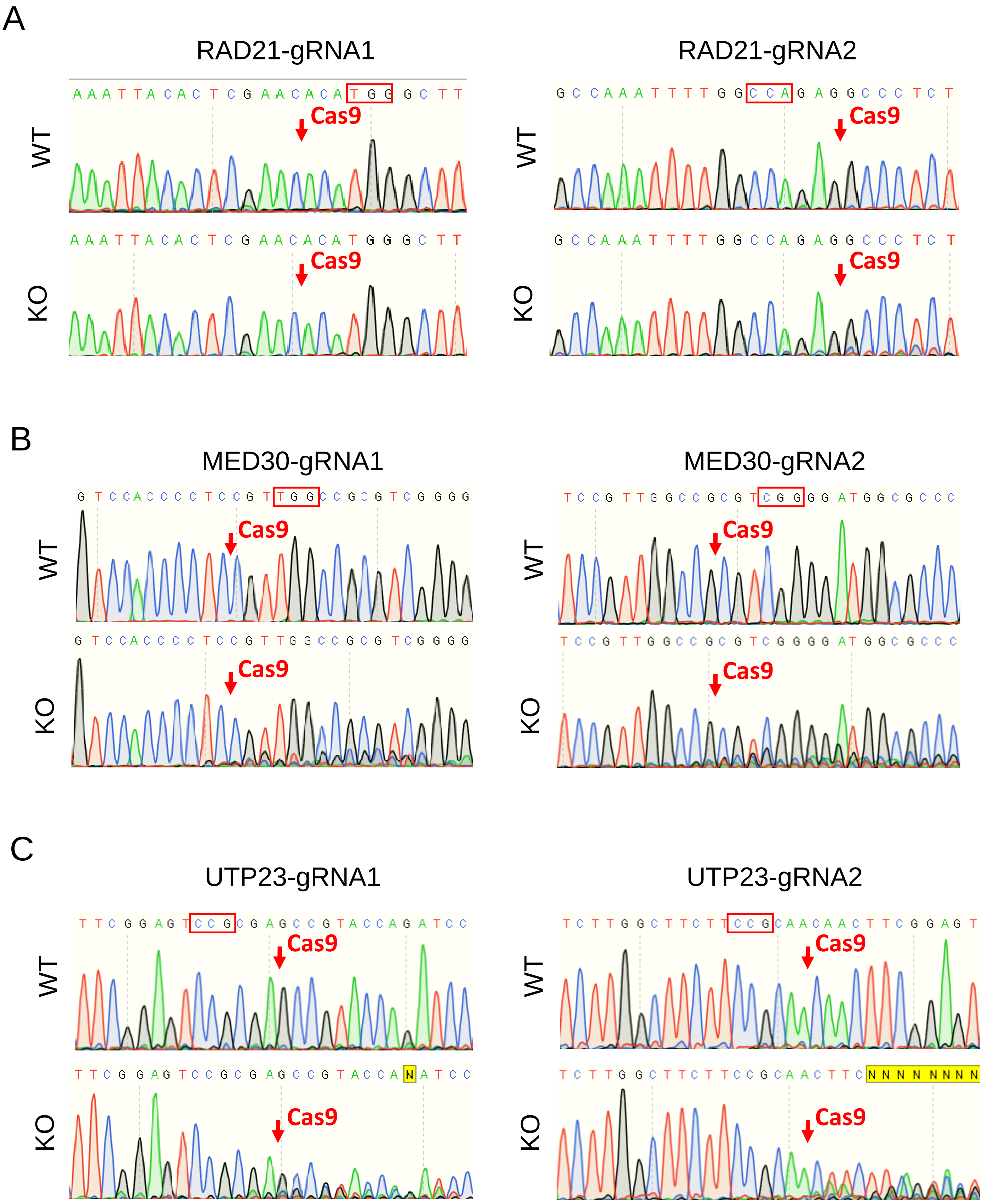
A
RAD21-gRNA1
WT
AAATTACACTCGAACACATGGGCTT
Cas9
KO
AAATTACACTCGAACACATGGGCTT
Cas9
RAD21-gRNA2
WT
GCCAAATTTTGGCCAGAGGCCCTCT
Cas9
KO
GCCAAATTTTGGCCAGAGGCCCTCT
Cas9
B
MED30-gRNA1
WT
GTCCACCCCTCCGTTGGCCGCGTCGGGG
Cas9
KO
GTCCACCCCTCCGTTGGCCGCGTCGGGG
Cas9
MED30-gRNA2
WT
TCCGTTGGCCGCGTCGGGGATGGCGCCC
Cas9
KO
TCCGTTGGCCGCGTCGGGGATGGCGCCC
Cas9
C
UTP23-gRNA1
WT
TTCGGAGTCCGCGAGCCGTACCAGATCC
Cas9
KO
TTCGGAGTCCGCGAGCCGTACCANATCC
Cas9
UTP23-gRNA2
WT
TCTTGGCTTCTTCCGCAACAACTTCGGAGT
Cas9
KO
TCTTGGCTTCTTCCGCAACTTCNNNNNNNN
Cas9

Figure S8

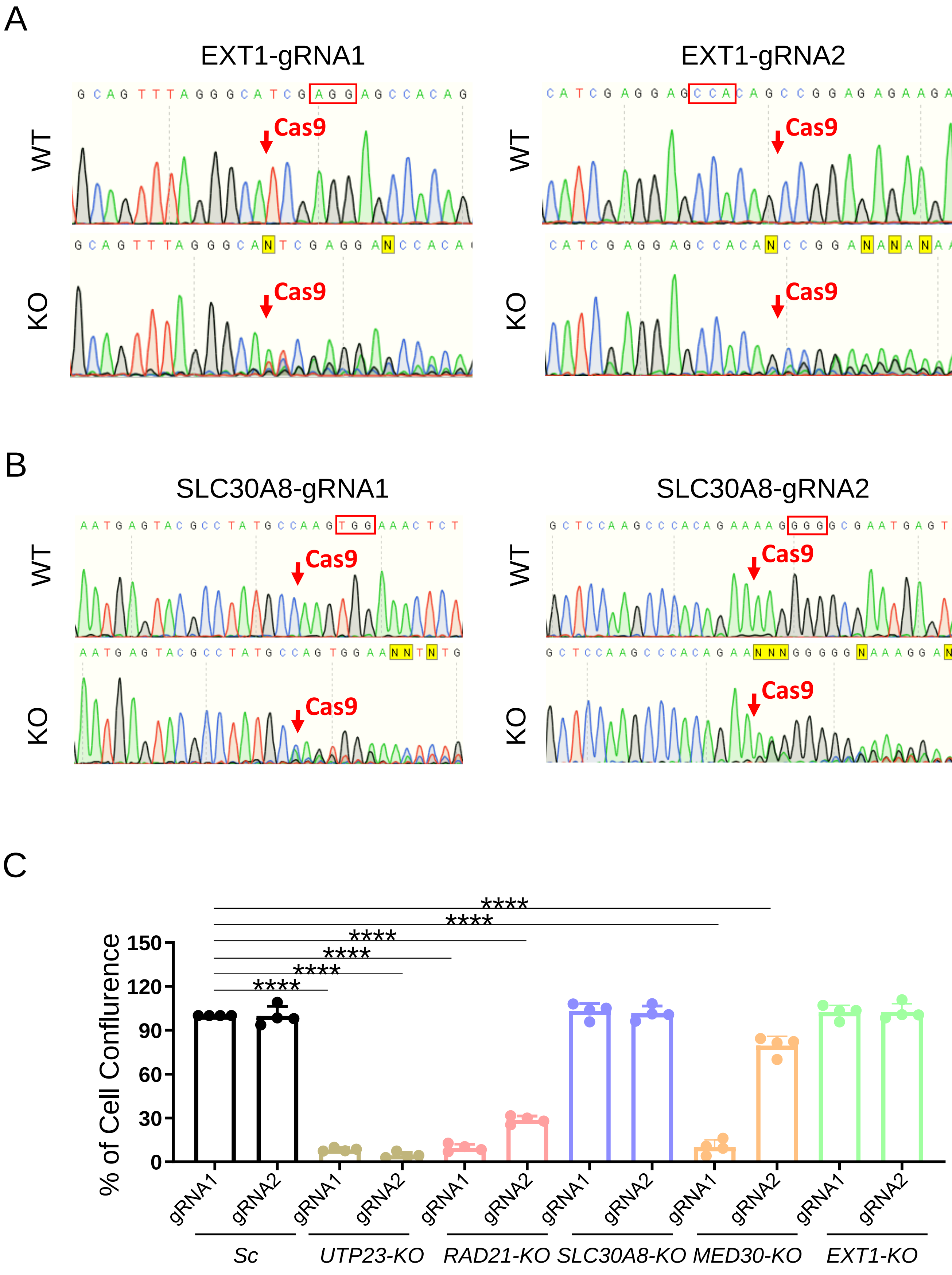
A
EXT1-gRNA1
EXT1-gRNA2
G C A G T T T A G G G C A T C G A G G A G C C A C A G
G C A G T T T A G G G C A N T C G A G G A N C C A C A
C A T C G A G G A G C C A C A G C C G G A G A G A A G A A
C A T C G A G G A G C C A C A N C C G G A N A N A N A A A
WT
KO
Cas9
B
SLC30A8-gRNA1
SLC30A8-gRNA2
A A T G A G T A C G C C T A T G C C A A G T G G A A A C T C T
A A T G A G T A C G C C T A T G C C A G T G G A A N N T N T G
G C T C C A A G C C C A C A G A A A A G G G G G C G A A T G A G T A C
G C T C C A A G C C C A C A G A A N N N G G G G G N A A A G G A N N C
WT
KO
Cas9
C
% of Cell Confluence
0
30
60
90
120
150
****
gRNA1
gRNA2
Sc
UTP23-KO
RAD21-KO
SLC30A8-KO
MED30-KO
EXT1-KO

Figure S9

A

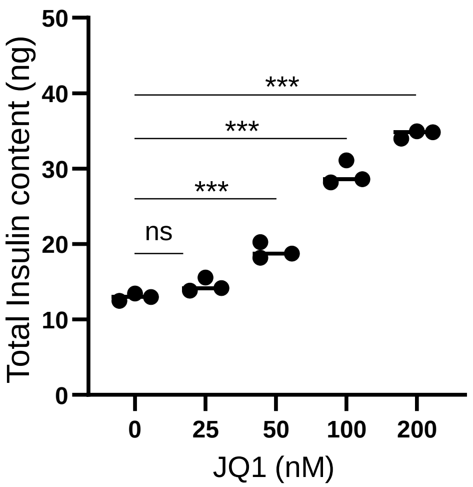

B

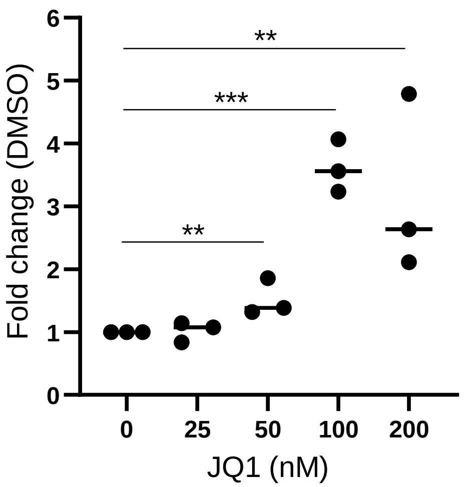

C

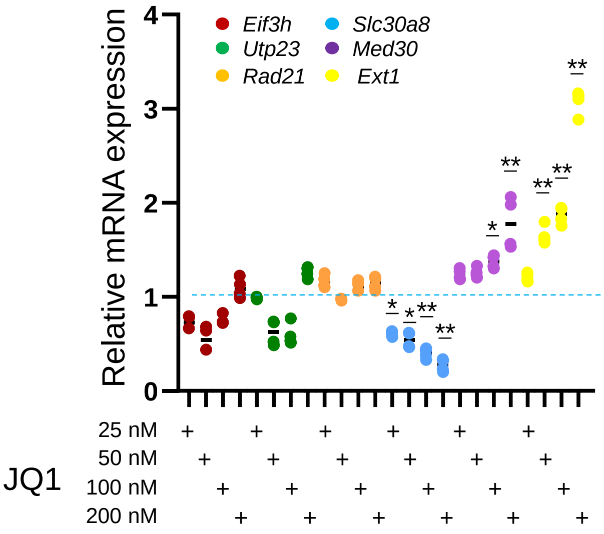

Figure S10
