# Supplementary figures and images for: Multiple genetic variants at the SLC30A8 locus affect local super-enhancer activity and influence pancreatic β-cell survival and function
Source: bioRxiv. 2023 Oct 11:2023.07.13.548906. Originally published 2023 Jul 13. Preprint. [Version 2] doi: 10.1101/2023.07.13.548906 (PMC10369998; doi:10.1101/2023.07.13.548906)

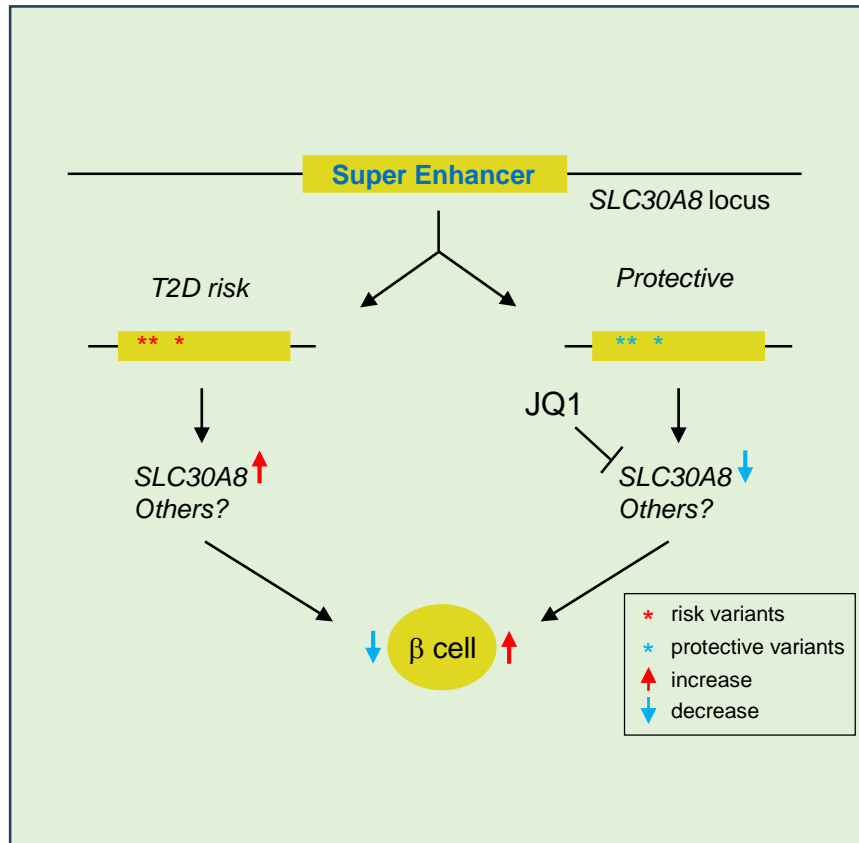

Supplement: Supplement 3 [file media-3.pdf]
