# Supplementary material for: Multiple genetic variants at the SLC30A8 locus affect a local super-enhancer cluster to influence pancreatic β-cell survival and function
Source: bioRxiv. 2023 Jul 13:2023.07.13.548906. Preprint. [Version 1] doi: 10.1101/2023.07.13.548906 (PMC10369998; doi:10.1101/2023.07.13.548906)
Supplement: Supplementary file 1 [file media-1.pdf]

Supplementary Information

Supplementary Figure legends

Figure S1 **GWAS identified genetic variants at the *SLC30A8* gene locus**. A. The GWAS variants (red dots) are located at the 3' end of the SLC30A8 gene. B. Islet-specific transcriptional factor binding sites at the SLC30A8 locus. R1 and R2 (shown in red) are two active enhancer regions bearing 3 GWAS variants. R1 and R2 in red are two enhancer regions bearing GWAS variants.

Figure S2 **Epigenomic map at the *SLC30A8* locus in all tissues and cells**. A-E. The data were extracted from EpiMap (http://compbio.mit.edu/epimap). Red box: islet-specific super-enhancer represented by strong regulome signals.

Figure S3 **Detailed locations of R1 and R2 regions at the *SLC30A8* locus**. R1 region bearing rs13266634 and rs3802177 is overlap with the exon8 of the *SLC30A8* gene while R2 bearing rs35859536 is downstream of the *SLC30A8* transcript.

Figure S4 **Role of R1 in β-cell function**. A. Diagram of R1 genomic DNA fragments in pGL4.23 luciferase vector. B and C. Promoter-luciferase assay in EndoC-βH3 cells. Data are mean ± SEM. *, $P < 0.05$; **, $P < 0.01$; ***, $P < 0.005$. $n = 3$. D. Sanger sequencing of PCR product amplified from R1-del1 cell. Red and blue bars represent the 5' and 3' end of DNA sequence flanking R1 region. It seems that the Cas9 cutting caused mutation(s) before ligation, which led to unclear DNA sequencing result. Primers used to amplify genomic DNA were listed in Table S3. E. Sanger sequencing of PCR product amplified from R1-del2 cell. Red and blue bars represent the 5' and 3' end of DNA sequence flanking R2 region. F. Diagram of qPCR strategy to determine the deletion efficiency. CXCL12 was served as an internal DNA copy number control. G. Deletion efficiency. The deletion efficiency rate (%) was determined by: 1. calculating the remaining wildtype allele (primer 1+4) of genomic DNA in R1-del cell and compared with wildtype allele in wild type control cell and 2. taking away the rate of DNA inversion (primers 1+3 or primers 2+3) in R1-del cell. $n = 2$.

Figure S5 **Role of R2 in β-cell function**. A. Diagram of R2 DNA fragments in pGL4.23 luciferase vector. B and C. Promoter-luciferase assay in EndoC-βH3 cells. Data are mean ± SEM. *, $P < 0.05$; **, $P < 0.01$; ***, $P < 0.005$. $n = 3$. D. Sanger sequencing of PCR product amplified from R2-del1 cell. E. Sanger sequencing of PCR product amplified from R2-del2 cell. F. Diagram of qPCR strategy to determine CRISPR-Cas9 mediated genomic DNA deletion efficiency. G. Deletion efficiency. $n = 2$.

Figure S6 **Analysis of individual variants in EndoC-βH3 cells**. A. Sanger sequencing confirmation of luciferase vectors carrying either risk or protective variant. B. Sanger sequencing analysis of PCR product amplified from genomic DNA region at variant site after CRISPR-Cas9 mediated genome editing. The editing resulted in mixed DNA sequences with no clear sequencing pattern at Cas9 cutting site in all three cell lines. C. Mutation efficiency of CRISPR-Cas9 mediated genome editing. The primer sets used for PCR amplification and DNA sequencing were listed in Table S3.

Figure S7 **Sanger sequencing of PCR product amplified from genomic DNA in wild-type and gene KO cells**. A. *MED30*. B. *UTP23*. C. *RAD21*. Note that the genomic DNAs were extracted from live cells. Since the knockout cells didn't survive well, the sequencing results may not represent the KO effects. Nevertheless, some changes were observed at the Cas9 cutting site: MED30-gRNA1: 1 bp (G) insertion; MED30-gRNA2: 2 bp (GC) deletion; UTP23-gRNA1: 1 bp (A) insertion and UTP23-gRNA2: 3 bp (CAA) deletion. No significant change was observed in both RAD21-KO cells.

Figure S8 **Sanger sequencing of PCR product amplified from genomic DNA in wild-type and gene KO cells**. A. *EXT1*. B. *SLC30A8*. Sequencing changes were identified at the Cas9 cutting site as follows: EXT1-gRNA1: 1 bp (A) insertion; EXT1-gRNA2: mixed with no clear pattern; SLC30A8-gRNA1: 1 bp (C) deletion and SLC30A8-gRNA2: 1 bp (A) deletion.

Supplementary Tables

Table S1 Key Resources

REAGENT or RESOURCE	SOURCE	IDENTIFIER
Bacteria and Virus Strain		
Stbl3 competent cells	Thermo Fisher Scientific	Cat# C737303
10-beta competent cells	New England Biolabs	Cat# C30191
Chemicals, Peptides, and Recombinant Proteins		
3-Isobutyl-1-methylxanthine (IBMX)	Sigma-Aldrich	Cat# I5879
Sodium Selenite	Sigma-Aldrich	Cat# S1382
Nicotinamide	Sigma-Aldrich	Cat# 481907
Paraformaldehyde	Agar Scientific	Cat# AGR1026
TRIzol	Invitrogen	Cat# 15596018
Albumin from Bovine Serum Fraction V	Roche Diagnostics	Cat# 10775835001
Human Transferrin	Sigma-Aldrich	Cat# T8158
ECM	Sigma-Aldrich	Cat# E1270
Critical Commercial Assay		
Dual-Luciferase Assay Reporter Assay System	Promega	Cat# E1910
Lipofectamine 2000	Thermo Fisher Scientific	Cat# 11668025
Insulin Ultra-sensitive Assay Kit	Cisbio Bioassays	Cat# 62IN2PEH
High-Capacity cDNA Reverse Transcription Kit	Thermo Fisher Scientific	Cat# 4368814
Taqman™ Fast advanced master mix	Thermo Fisher Scientific	Cat# 4444553
Fast SYBR™ Green Master Mix	Thermo Fisher Scientific	Cat# 4385612
Phusion High fidelity DNA polymerase	Thermo Fisher Scientific	Cat# F530
Experimental Model: cell line		
EndoC-βH3	Humancelldesign.com	N/A
Oligonucleotides		
gRNAs	See Table S2	
Genomic DNA amplification	See Table S3	
SYBR™ green qPCR for gene	See Table S4	
Luciferase assay	See Table S5	
Recombinant DNA		
pMD2.G	Didier Trono (https://tronolab.epfl.ch)	Cat# 12259; RRID: Addgene_12259

pSPAX2	Didier Trono (https://tronolab.epfl.ch)	Cat# 12260; RRID: Addgene_12260
pLenti-CRISPR-RIP-Cas9	Hu, M. et al., Cell Reports (2021)	N/A
pGL4.23	Promega	E8411
pRL-Renilla	Promega	E2231
Taqman^{TM} qPCR primer/probe		
SLC30A8	Thermo Fisher Scientific	Cat# 4331182; Assay ID: Hs00545183_ml
UTP23	Thermo Fisher Scientific	Cat# 4331182; Assay ID: Hs00260536_ml
RAD21	Thermo Fisher Scientific	Cat# 4331182; Assay ID: Hs00366726_ml
MED30	Thermo Fisher Scientific	Cat# 4331182; Assay ID: HS00369804_ml
EXT1	Thermo Fisher Scientific	Cat# 4331182; Assay ID: Hs00609162_ml
ACTB	Thermo Fisher Scientific	Cat# 4331182; Assay ID: Hs01060665_gl
EIF3H	Thermo Fisher Scientific	Cat# 4331182; Assay ID: Hs00186779_m1
INS	Thermo Fisher Scientific	Cat# 4331182; Assay ID: Hs00355773_m1
MAL2	Thermo Fisher Scientific	Cat# 4351372; Assay ID: Hs01043579_u1
ENPP2	Thermo Fisher Scientific	Cat# 4331182; Assay ID: Hs00905117_m1
SYBU	Thermo Fisher Scientific	Cat# 4331182; Assay ID: Hs01052028_m1
Software		
GraphPad Prism 9	GraphPad Software	RRID: SCR_002798
Illustrator	Adobe	RRID:SCR_010279
Photoshop	Adobe	RRID: SCR_014199

Table S2 Guide RNA (gRNA) sequences for CRISPR-Cas9 genome editing

SCRAMBLED-gRNA1	GAACTCAACCAGAGGGCCAA
SCRAMBLED-gRNA2	GGGAGGTGGCTTTAGGTTTT
SLC30A8-gRNA1	GCTCCAAGCCCACAGAAAAG
SLC30A8-gRNA2	ATGAGTACGCCTATGCCAAG
RAD21-gRNA1	CTAAATTACACTCGAACACA
RAD21-gRNA2	TCTCAGTAAAAGAGGGCCTC
UTP23-gRNA1	GCAGGATCTGGTACGGCTCG
UTP23-gRNA2	CGCGGACTCCGAAGTTGTTG
MED30-gRNA1	CCCCGACGCGGCCAACGGAG
MED30-gRNA2	CACCCCTCCGTTGGCCGCGT
EXT1-gRNA1	GCTTGCAGTTTAGGGCATCG
EXT1-gRNA2	TGTGTTCTTCTCTCCGGCTG
R1-gRNA1	GGGGCTTAGGTCTGACTTGA
R1-gRNA-2	GCCTCTTCCTTCATGGTGAA
R1-gRNA3	TTGTCTTTGACCTGCTGGGG
R2-gRNA3	GGGGAGGATGCCTATAAGAT

R2-gRNA-2	GTTTGGGGTAAACTGCAAAC
R2-gRNA-3	TTGCTGGAGTGGTGTACATG
rs13266634-gRNA	AACCACTTGGCTGTCCCGGC
rs3802177-gRNA	AAAGGAAGAAATTCATGTCA
rs35859536-gRNA	TGGTAGATGGCTAGGTACGA

Table S3 Primer set for PCR amplification of genomic DNA

gSLC30A8-F1	AGCGGTTCCAAGTATGGGCAGG
gSLC30A8-F2	GCAGGGTGGGTCAGTGAGTGG
gSLC30A8-R	GAAAGACTCACCCACGACCTCTGC
gRAD21-F1	CTCTAGCTCTTGGCCAGTAAACAGAC
gRAD21-F2	TGACTCTTGTCCCTTTTACTTTCAGCTC
gRAD21-R	CTTGCACTCCAATGCCCCTACC
gUTP23-F1	GCTTCATTTCCGGGTGAAACTGGC
gUTP23-F2	GTGAAACTGGCATTGAGGGTACTGG
gUTP23-R	CGCAATGAAGGTCTGTGAGCAACG
gMED30-F1	GCGGCCGCTGTTTTGAAATCG
gMED30-F2	GGGGTCTCTCAAGCTGGTTCC
gMED30-R	CTTTCCCTCCCAGCTGCATCC
gEXT1-F1	GAAGTCTTTACAGGCGGGAAGATGG
gEXT1-F2	GGGAAGATGGCGGACTGGAGC
gEXT1-R	ACTCCATGCGGCACTTCTTGC
R1-KO-F1	AATTATGGTGGGGTCCCACACTG
R1-KO-F2	GGCAGTGGGAGGGCATTTACTGC
R1-KO-R2	CTGTTACTTCGGCTCCACTCAGG
R2-KO-F1	TTTTCCTGTTTATTCCAGAACTTGGTAGG
R2-KO-F2	CTCTCATTACTGAGATGTCAGTTTCTGC
R2-KO-R2	CCTGGGGGAAGCTACAGTGG

Table S4 SYBR^{TM} Green qPCR primer for lncRNA expression and genotyping

β-ACTIN-F	CACCATTGGCAATGAGCGGTTC
β-ACTIN-R	AGGTCTTTGCGGATGTCCACGT
RAD21AS1-F	CTGTTTGTGGAGTCCACAACTGAGC
RAD21AS1-R	GTGTCCTCTTCAATTTCTTTCATCACTGG
RP11-654G14-F	AATATGCTCCCTGTGTTATTATTGCC
RP11-654G14-R	CCTAAACTCTCGACCAAGTCACC
rs13266634-F	GTCAGAGCAGTCGCCCATGC
rs13266634-R	CCACTTGGCTGTCCCGGCTGG
rs3802177-F	ACATGCTGCTATGCAGTTTCTGC
rs3802177-R	AAATGTGCATTGCACCATGACATG
rs35859536-F	GGTCGTGTGCCTGGTTCTAGACG
rs35859536-R	ATGCCAGATTGCCTCCATCGTAC
qR1-1	CATTTGAAAACTGTGCTTTCTCAGACATCG
qR1-2	CAAACGTGGCTTCCTCTGAGTGC
qR1-3	GGAAGAAATTCATGTCATGGTGCAATGC
qR1-4	TCCAATTGATTGATGGATCTCAGTGC
qR2-1	CGGAAGTAGAGCCAAGAAAGATTAGAGG
qR2-2	CTCAGAGAGAGAGGAGAGAATTGCTGG
qR2-3	CAGCACTTCATTCTCTTTTCCTGCAG
qR2-4	TGTGATGAGGCTGGTTCTTTGAGTTTGG

Table S5 Primer set for DNA cloning into pGL4.23

rs13266634-Luc-KpnI	ATGTCAGGTACCGTCAGAGCAGTCGCCCATGC
rs13266634-Luc-XhoI	AATCATCTCGAGCTGAATGGTGAGTGAGTGCATCG
rs3802177-Luc-KpnI	ATGTCAGGTACCGACTAGCTCAGTCACACCGTCAG
rs3802177-Luc-XhoI	AATCATCTCGAGCACATTCCTGCAATTGAACACAGC
rs35859536-Luc-KpnI	ATGTCAGGTACCGTCGTGTGCCTGGTTCTAGACG
rs35859536-Luc-XhoI	AATCATCTCGAGGATGACGATTTCACTCTTGCTCC
R1-Luc-KpnI-F1	ATGTCAGGTACCGGGGCTTAGGTCTGACTTGAGG
R1-Luc-KpnI-F2	ATGTCAGGTACCCAGAGATCCCTGGGAGCTGG
R2-Luc-KpnI-F1	ATGTCAGGTACCCTGCTAGTGCTGGAATAGATCTCGG
R2-Luc-XhoI-R	AATCATCTCGAGTGTGATGAGGCTGGTTCTTTGAGTTTGG

ABBREVIATIONS

T2D, Type 2 Diabetes

GWAS, Genome-wide association study

R, Regulatory region

SNP, Single-nucleotide polymorphism

CHIP-seq: Chromatin immunoprecipitation and DNA sequencing

ATAC-seq, Assay for Transposase-Accessible Chromatin using sequencing

3C, Chromosome Conformation Capture

4C, Circular Chromosome Conformation Capture

CTCF, CCCTC-bind factor

pcHi-C, Promoter Capture Hi-C

TAD, Topologically Associating Domain

SLC30A8, Solute Carrier Family 30 Member 8

RAD21, Double-strand-break repair protein rad21 homolog

UTP23, UTP23 Small Subunit Processome Component

MED30, Mediator of RNA Polymerase II Transcription Subunit 30

EXT1, Exostosin Glycosyltransferase 1

CRISPR, Clustered Regularly Interspaced Short Palindromic Repeats

Cas9, Endonuclease from *Streptococcus pyogenes*

GSIS, Glucose-stimulated insulin secretion
