# Supplementary material for: Multiple genetic variants at the SLC30A8 locus affect a local super-enhancer cluster to influence pancreatic β-cell survival and function
Source: bioRxiv. 2023 Jul 13:2023.07.13.548906. Preprint. [Version 1] doi: 10.1101/2023.07.13.548906 (PMC10369998; doi:10.1101/2023.07.13.548906)
Supplement: Supplementary file 2 [file media-2.pdf]

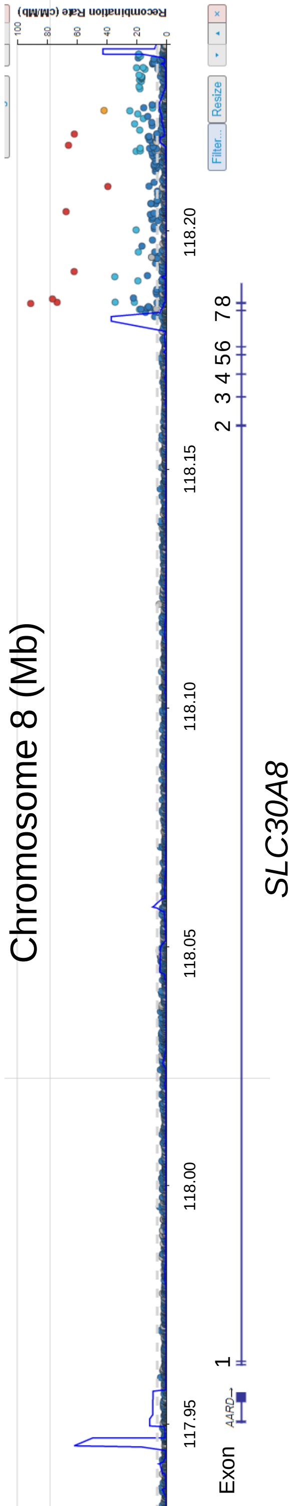

Chromosome 8 (Mb)
-log10(p)
Recombination Rate (cM/Mb)
117.95
118.00
118.05
118.10
118.15
118.20
Exon
AARD
1
2 3 4 5 6 7 8
SLC30A8
Filter...
Resize

Figure S1

A

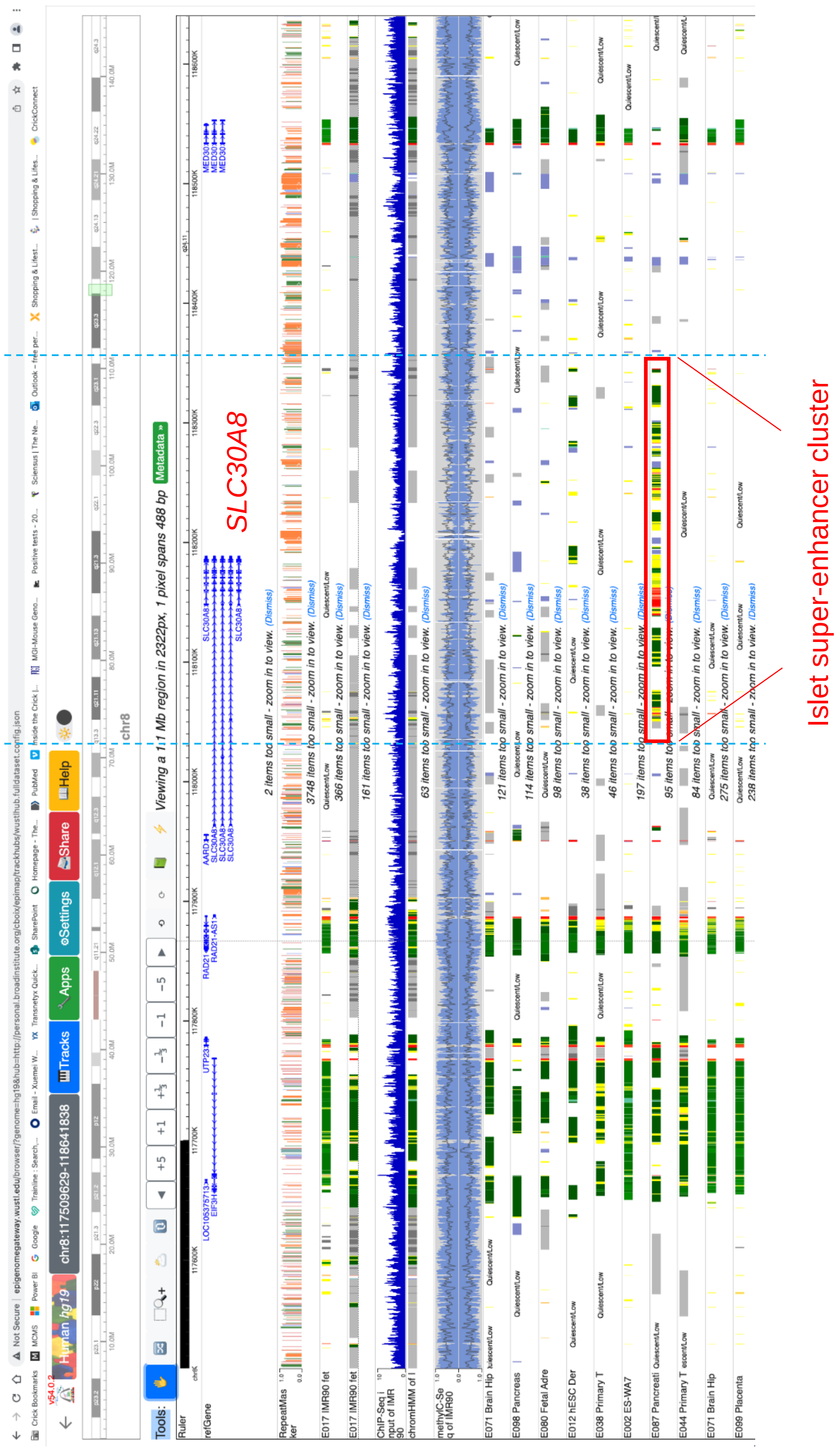

SLC30A8
Islet super-enhancer cluster
Viewing a 1.1 Mb region in 2322px, 1 pixel spans 488 bp

Figure S2

B

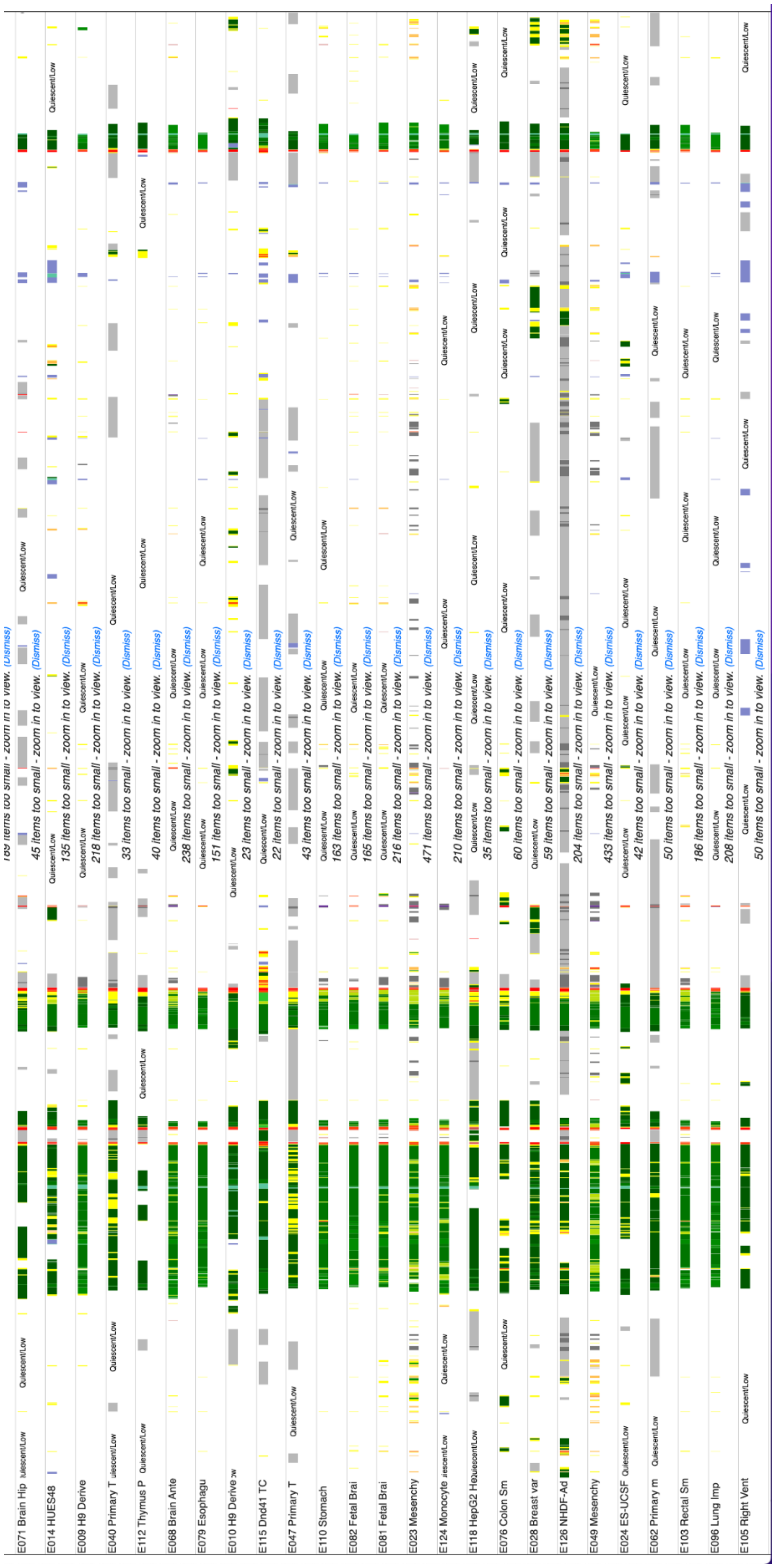
E071 Brain Hip
E014 HUES48
E009 H9 Derive
E040 Primary T
E112 Thymus P
E068 Brain Ante
E079 Esophagu
E010 H9 Derive
E115 Dnd41 TC
E047 Primary T
E110 Stomach
E082 Fetal Brai
E081 Fetal Brai
E023 Mesenchy
E124 Monocyte
E118 HepG2 He
E076 Colon Sm
E028 Breast var
E126 NHDF-Ad
E049 Mesenchy
E024 ES-UCSF
E062 Primary m
E103 Rectal Sm
E096 Lung Imp
E105 Right Vent

Figure S2

C

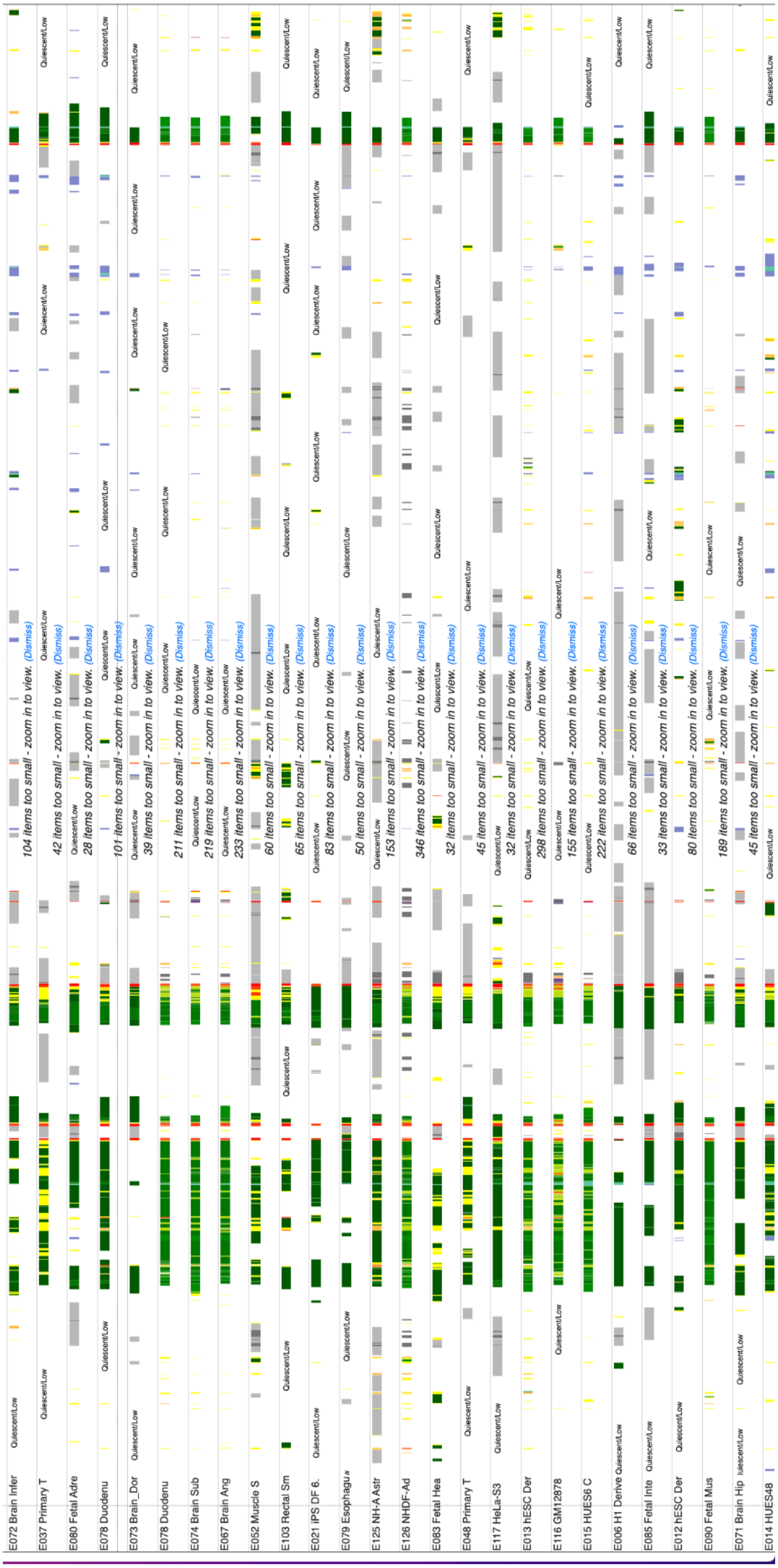
E072 Brain Infer
E037 Primary T
E080 Fetal Adre
E078 Duodenu
E073 Brain_Dor
E078 Duodenu
E074 Brain Sub
E067 Brain Ang
E052 Muscle S
E103 Rectal Sm
E021 iPS DF 6.
E079 Esophagu
E125 NH-A Astr
E126 NHDF-Ad
E083 Fetal Hea
E048 Primary T
E117 HeLa-S3
E013 hESC Der
E116 GM12878
E015 HUES6 C
E006 H1 Derive
E085 Fetal Inte
E012 hESC Der
E090 Fetal Mus
E071 Brain Hip
E014 HUES48
Quiescent/Low
104 items too small - zoom in to view. (Dismiss)
42 items too small - zoom in to view. (Dismiss)
28 items too small - zoom in to view. (Dismiss)
101 items too small - zoom in to view. (Dismiss)
39 items too small - zoom in to view. (Dismiss)
211 items too small - zoom in to view. (Dismiss)
219 items too small - zoom in to view. (Dismiss)
233 items too small - zoom in to view. (Dismiss)
60 items too small - zoom in to view. (Dismiss)
65 items too small - zoom in to view. (Dismiss)
83 items too small - zoom in to view. (Dismiss)
50 items too small - zoom in to view. (Dismiss)
153 items too small - zoom in to view. (Dismiss)
346 items too small - zoom in to view. (Dismiss)
32 items too small - zoom in to view. (Dismiss)
45 items too small - zoom in to view. (Dismiss)
32 items too small - zoom in to view. (Dismiss)
298 items too small - zoom in to view. (Dismiss)
155 items too small - zoom in to view. (Dismiss)
222 items too small - zoom in to view. (Dismiss)
66 items too small - zoom in to view. (Dismiss)
33 items too small - zoom in to view. (Dismiss)
80 items too small - zoom in to view. (Dismiss)
189 items too small - zoom in to view. (Dismiss)
45 items too small - zoom in to view. (Dismiss)

Figure S2

D

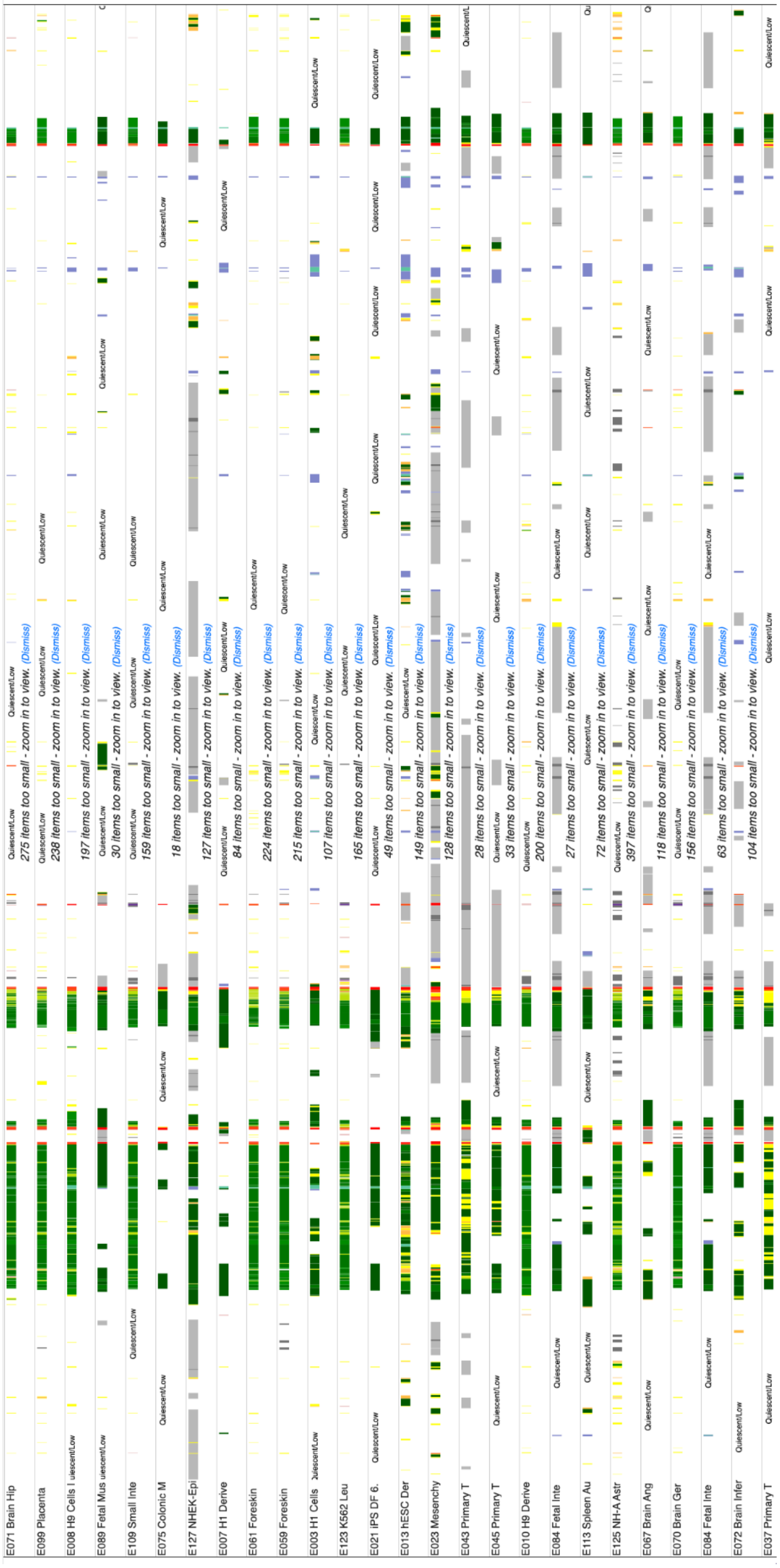
E071 Brain Hip
E099 Placenta
E008 H9 Cells I
E089 Fetal Mus
E109 Small Inte
E075 Colonic M
E127 NHEK-Epi
E007 H1 Derive
E061 Foreskin
E059 Foreskin
E003 H1 Cells
E123 K562 Leu
E021 iPS DF 6.
E013 hESC Der
E023 Mesenchy
E043 Primary T
E045 Primary T
E010 H9 Derive
E084 Fetal Inte
E113 Spleen Au
E125 NH-A Astr
E067 Brain Ang
E070 Brain Ger
E084 Fetal Inte
E072 Brain Infer
E037 Primary T
Quiescent/Low
275 items too small - zoom in to view. (Dismiss)
238 items too small - zoom in to view. (Dismiss)
197 items too small - zoom in to view. (Dismiss)
30 items too small - zoom in to view. (Dismiss)
159 items too small - zoom in to view. (Dismiss)
18 items too small - zoom in to view. (Dismiss)
127 items too small - zoom in to view. (Dismiss)
84 items too small - zoom in to view. (Dismiss)
224 items too small - zoom in to view. (Dismiss)
215 items too small - zoom in to view. (Dismiss)
107 items too small - zoom in to view. (Dismiss)
165 items too small - zoom in to view. (Dismiss)
49 items too small - zoom in to view. (Dismiss)
149 items too small - zoom in to view. (Dismiss)
128 items too small - zoom in to view. (Dismiss)
28 items too small - zoom in to view. (Dismiss)
33 items too small - zoom in to view. (Dismiss)
200 items too small - zoom in to view. (Dismiss)
27 items too small - zoom in to view. (Dismiss)
72 items too small - zoom in to view. (Dismiss)
397 items too small - zoom in to view. (Dismiss)
118 items too small - zoom in to view. (Dismiss)
156 items too small - zoom in to view. (Dismiss)
63 items too small - zoom in to view. (Dismiss)
104 items too small - zoom in to view. (Dismiss)

Figure S2

E

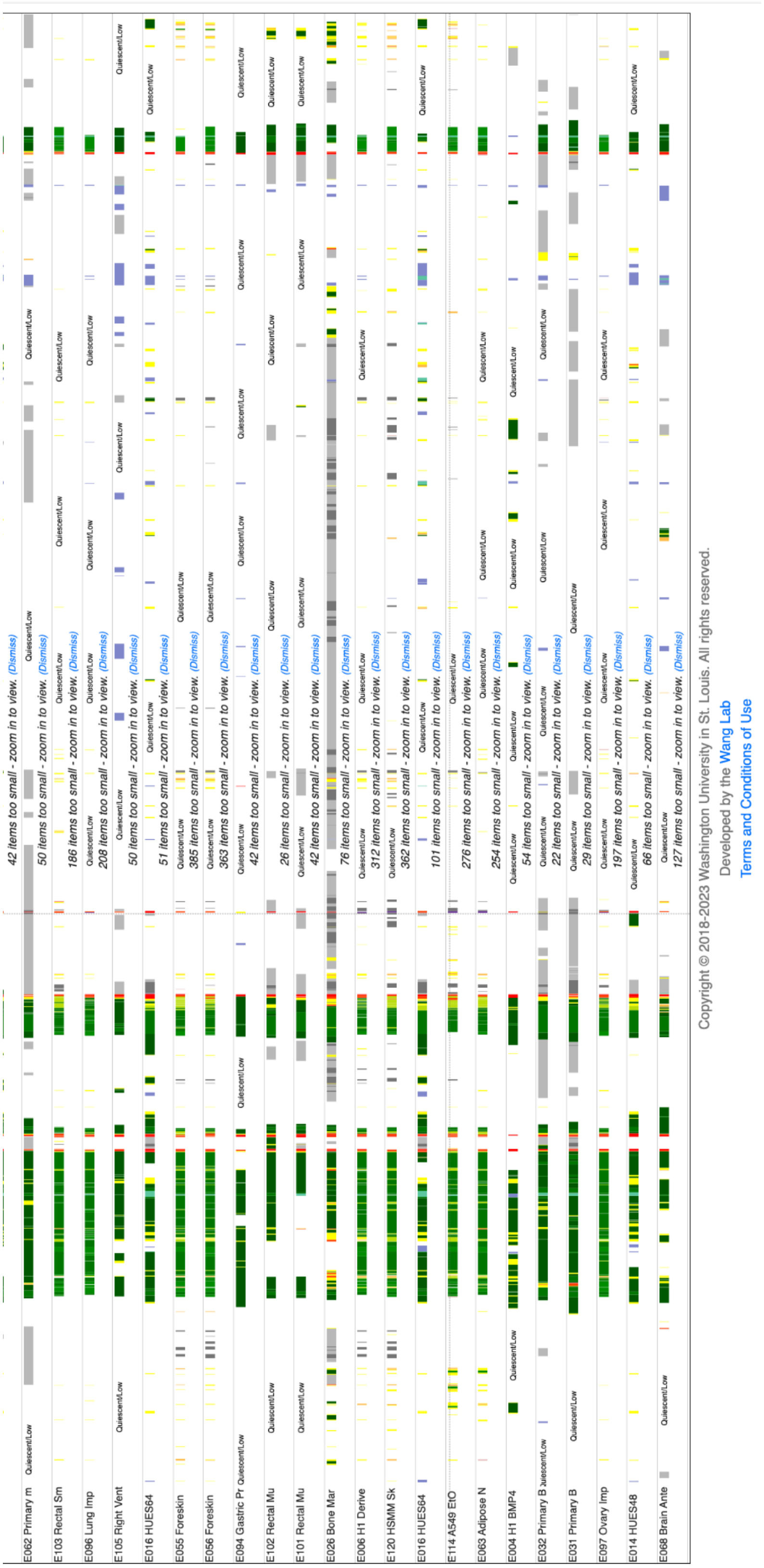
E062 Primary m
E103 Rectal Sm
E096 Lung Imp
E105 Right Vent
E016 HUES64
E055 Foreskin
E056 Foreskin
E094 Gastric Pr
E102 Rectal Mu
E101 Rectal Mu
E026 Bone Mar
E006 H1 Derive
E120 HSMM Sk
E016 HUES64
E114 A549 EtO
E063 Adipose N
E004 H1 BMP4
E032 Primary B
E031 Primary B
E097 Ovary Imp
E014 HUES48
E068 Brain Ante
Copyright © 2018-2023 Washington University in St. Louis. All rights reserved.
Developed by the Wang Lab
Terms and Conditions of Use

Figure S2

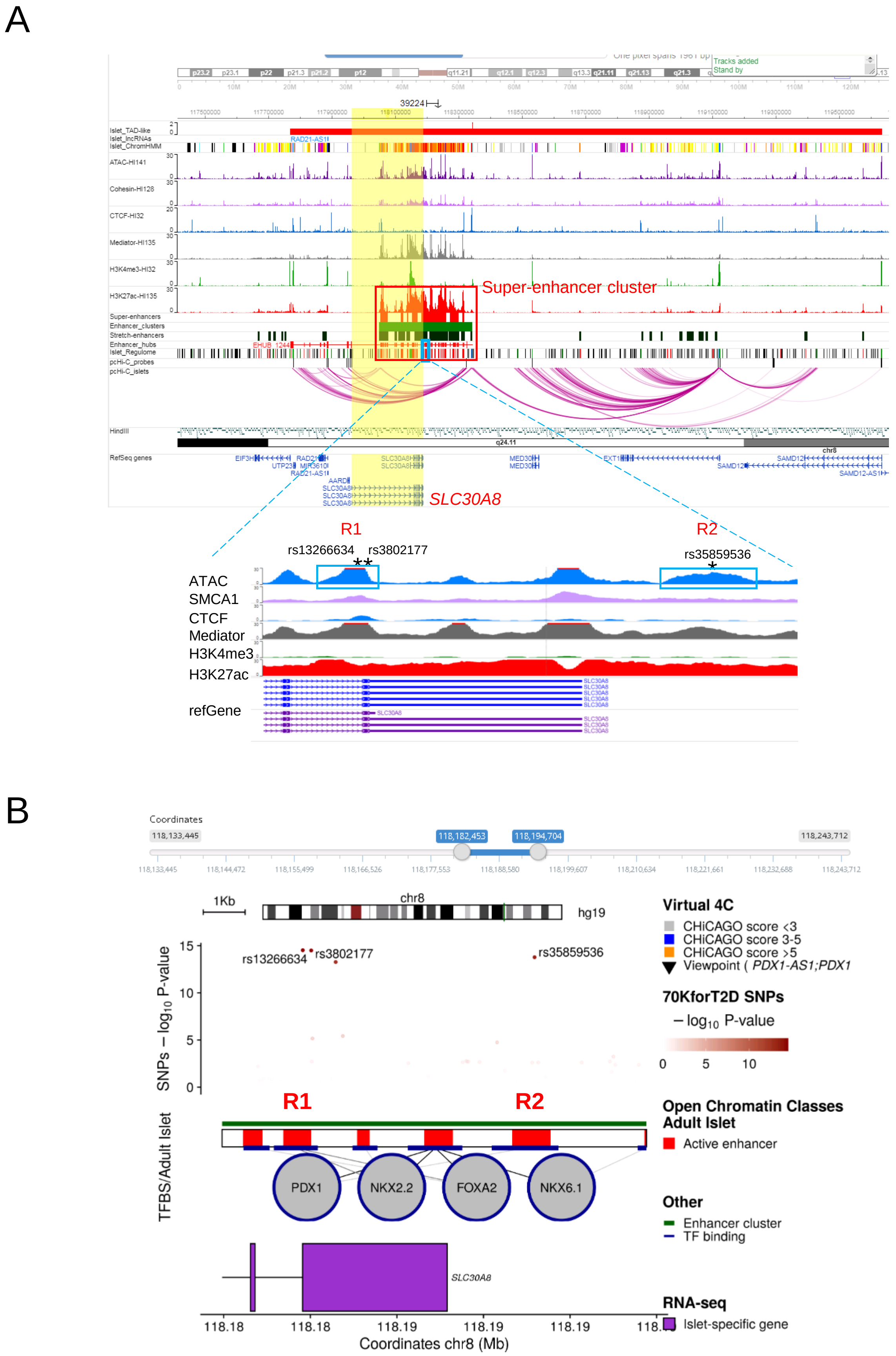
A
Super-enhancer cluster
SLC30A8
R1
R2
rs13266634
rs3802177
rs35859536
ATAC
SMCA1
CTCF
Mediator
H3K4me3
H3K27ac
refGene
B
Coordinates
118,133,445
118,182,453
118,194,704
118,243,712
1Kb
chr8
hg19
Virtual 4C
CHiCAGO score <3
CHiCAGO score 3-5
CHiCAGO score >5
Viewpoint (PDX1-AS1;PDX1
70KforT2D SNPs
SNPs – log10 P-value
R1
R2
TFBS/Adult Islet
PDX1
NKX2.2
FOXA2
NKX6.1
Open Chromatin Classes
Adult Islet
Active enhancer
Other
Enhancer cluster
TF binding
RNA-seq
Islet-specific gene
SLC30A8
Coordinates chr8 (Mb)

Figure S3

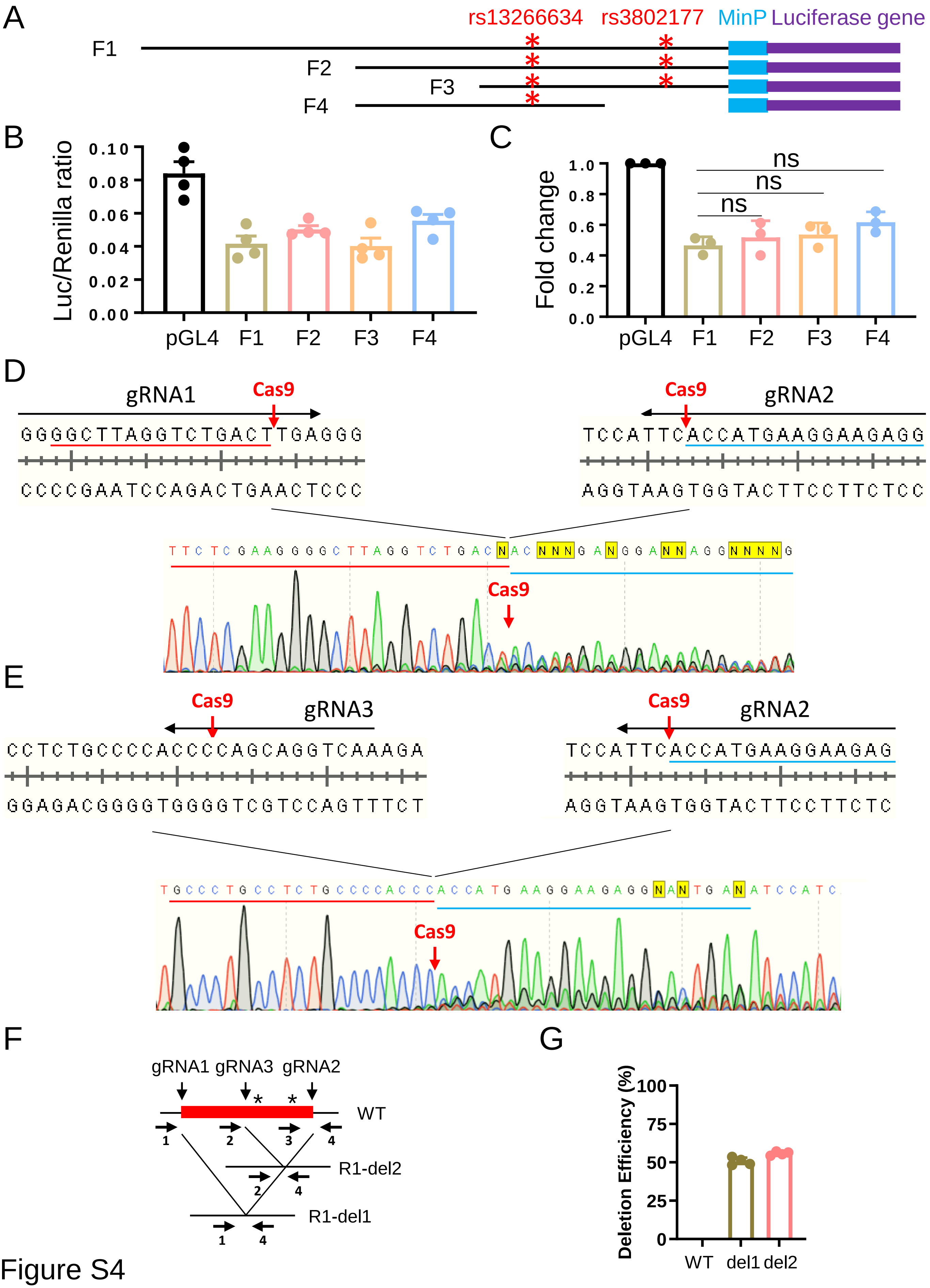
A
rs13266634
rs3802177
MinP
Luciferase gene
F1
F2
F3
F4
B
Luc/Renilla ratio
0.00
0.02
0.04
0.06
0.08
0.10
pGL4
F1
F2
F3
F4
C
Fold change
0.0
0.2
0.4
0.6
0.8
1.0
ns
ns
ns
pGL4
F1
F2
F3
F4
D
gRNA1
Cas9
GGGGCTTAGGTCTGACTTGAGGG
CCCCGAATCCAGACTGAACTCCC
Cas9
gRNA2
TCCATTCACCATGAAGGAAGAGG
AGGTAAGTGGTACTTCCTTCTCC
TTCTCGAAGGGGCTTAGGTCTGACNACNNNGANGGANNAGGNNNNG
Cas9
E
Cas9
gRNA3
CCTCTGCCCCACCCCAGCAGGTCAAAGA
GGAGACGGGGTGGGGTCGTCCAGTTTCT
Cas9
gRNA2
TCCATTCACCATGAAGGAAGAG
AGGTAAGTGGTACTTCCTTCTC
TGCCCTGCCTCTGCCCCACCCACCATGAAGGAAGAGGNANTGANATCCATC
Cas9
F
gRNA1 gRNA3 gRNA2
WT
1
2
3
4
R1-del2
2
4
R1-del1
1
4
G
Deletion Efficiency (%)
0
25
50
75
100
WT
del1
del2

Figure S4

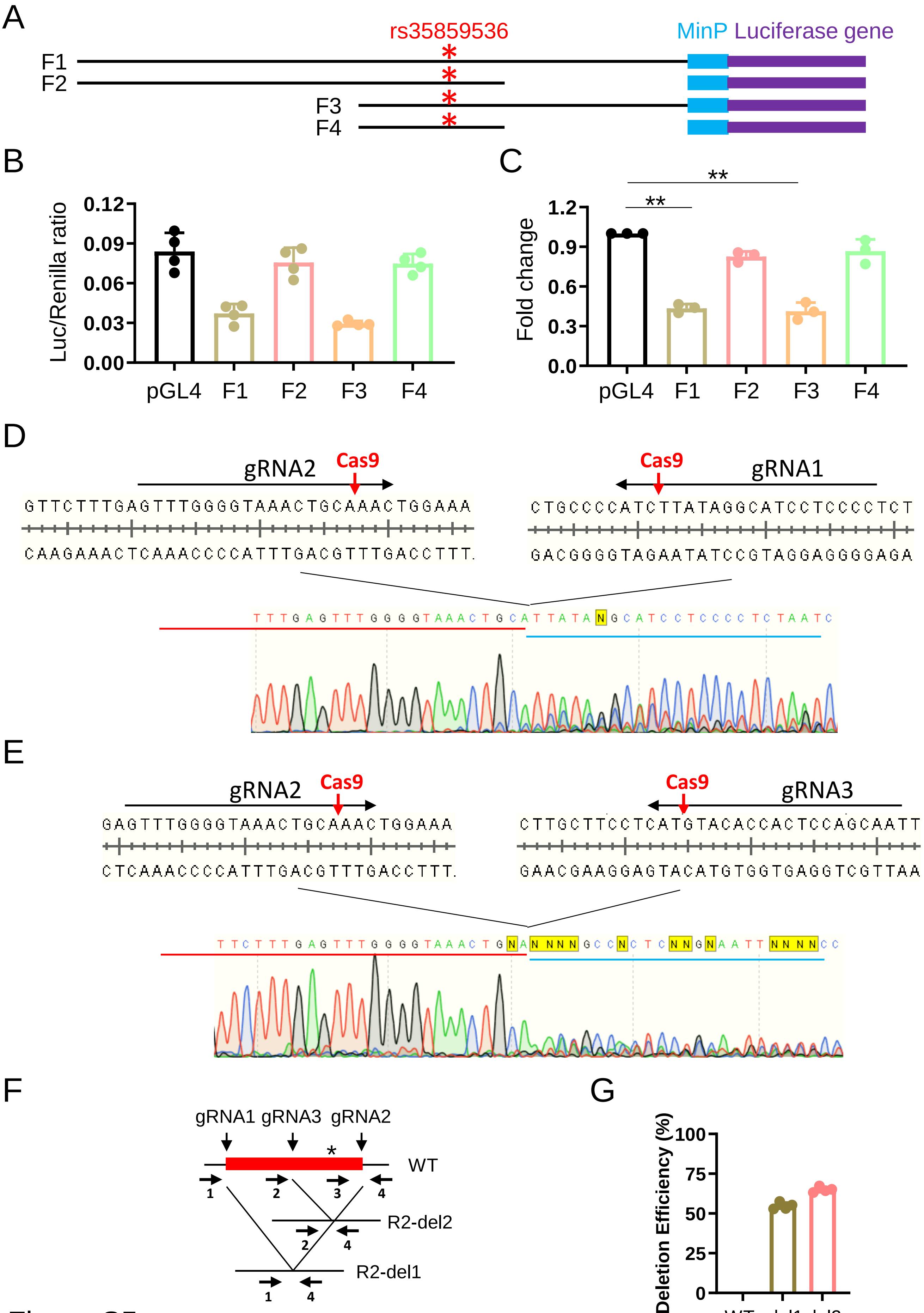
A
rs35859536
MinP Luciferase gene
F1
F2
F3
F4
B
Luc/Renilla ratio
0.12
0.09
0.06
0.03
0.00
pGL4 F1 F2 F3 F4
C
Fold change
1.2
0.9
0.6
0.3
0.0
**
**
pGL4 F1 F2 F3 F4
D
gRNA2
Cas9
Cas9
gRNA1
GTTCTTTGAGTTTGGGGTAAACTGCAAACTGGAAA
CAAGAAACTCAAACCCCATTTGACGTTTGACCTTT
CTGCCCCATCTTATAGGCATCCTCCCCTCT
GACGGGGTAGAATATCCGTAGGAGGGGAGA
TTTGAGTTTGGGGTAAACTGCATTATANGCATCCTCCCCTCTAATC
E
gRNA2
Cas9
Cas9
gRNA3
GAGTTTGGGGTAAACTGCAAACTGGAAA
CTCAAACCCCATTTGACGTTTGACCTTT
CTTGCTTCCTCATGTACACCACTCCAGCAATT
GAACGAAGGAGTACATGTGGTGAGGTCGTTAA
TTCTTTGAGTTTGGGGTAAACTGNANNNNGCCNCTCNNGNAATTNNNNCC
F
gRNA1 gRNA3 gRNA2
*
WT
1 2 3 4
R2-del2
2 4
R2-del1
1 4
G
Deletion Efficiency (%)
100
75
50
25
0
WT del1 del2

Figure S5

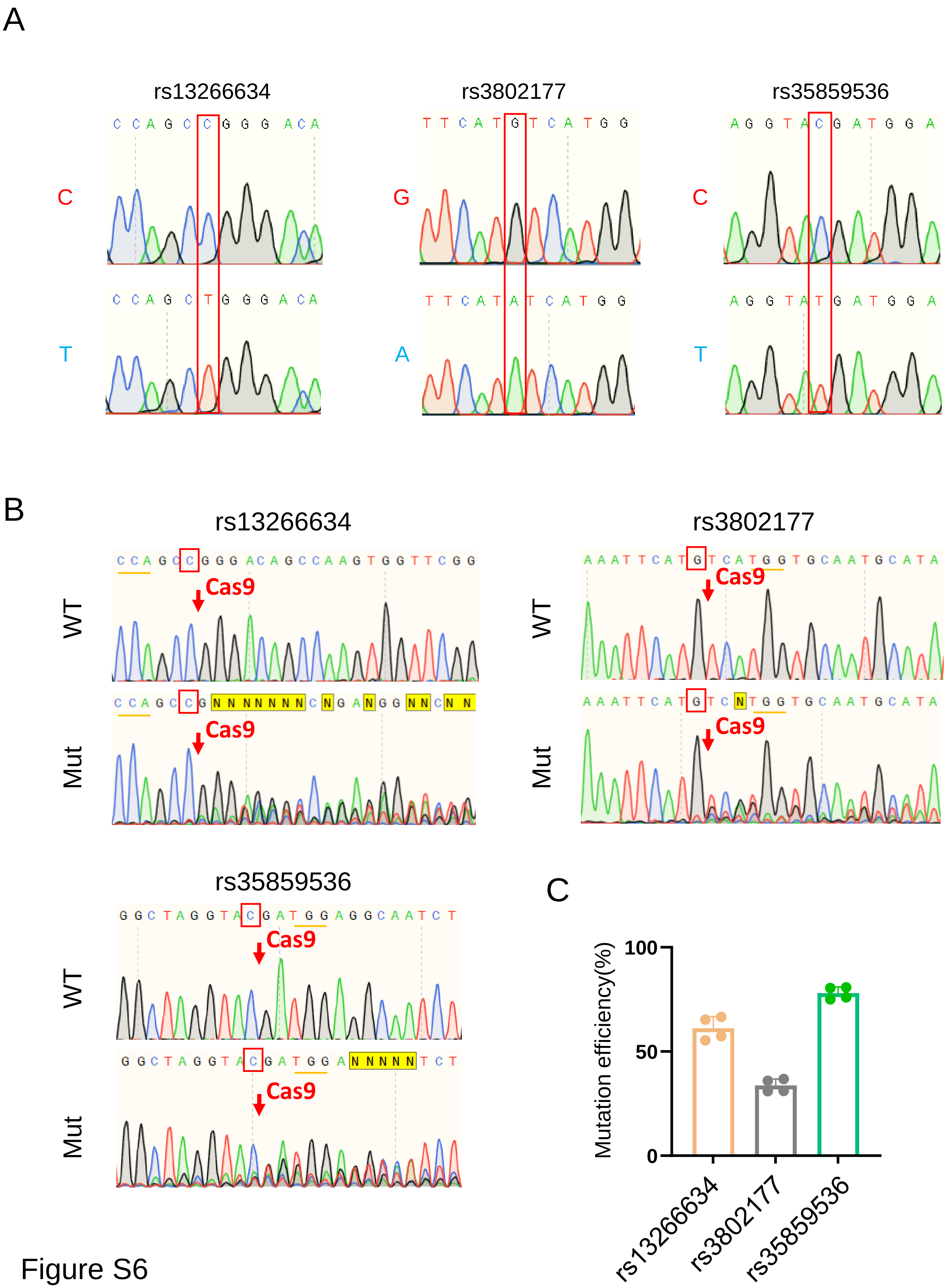
A
rs13266634
rs3802177
rs35859536
C
T
G
A
C
T
B
rs13266634
WT
Mut
Cas9
rs3802177
WT
Mut
Cas9
rs35859536
WT
Mut
Cas9
C
Mutation efficiency(%)
100
50
0
rs13266634
rs3802177
rs35859536

Figure S6

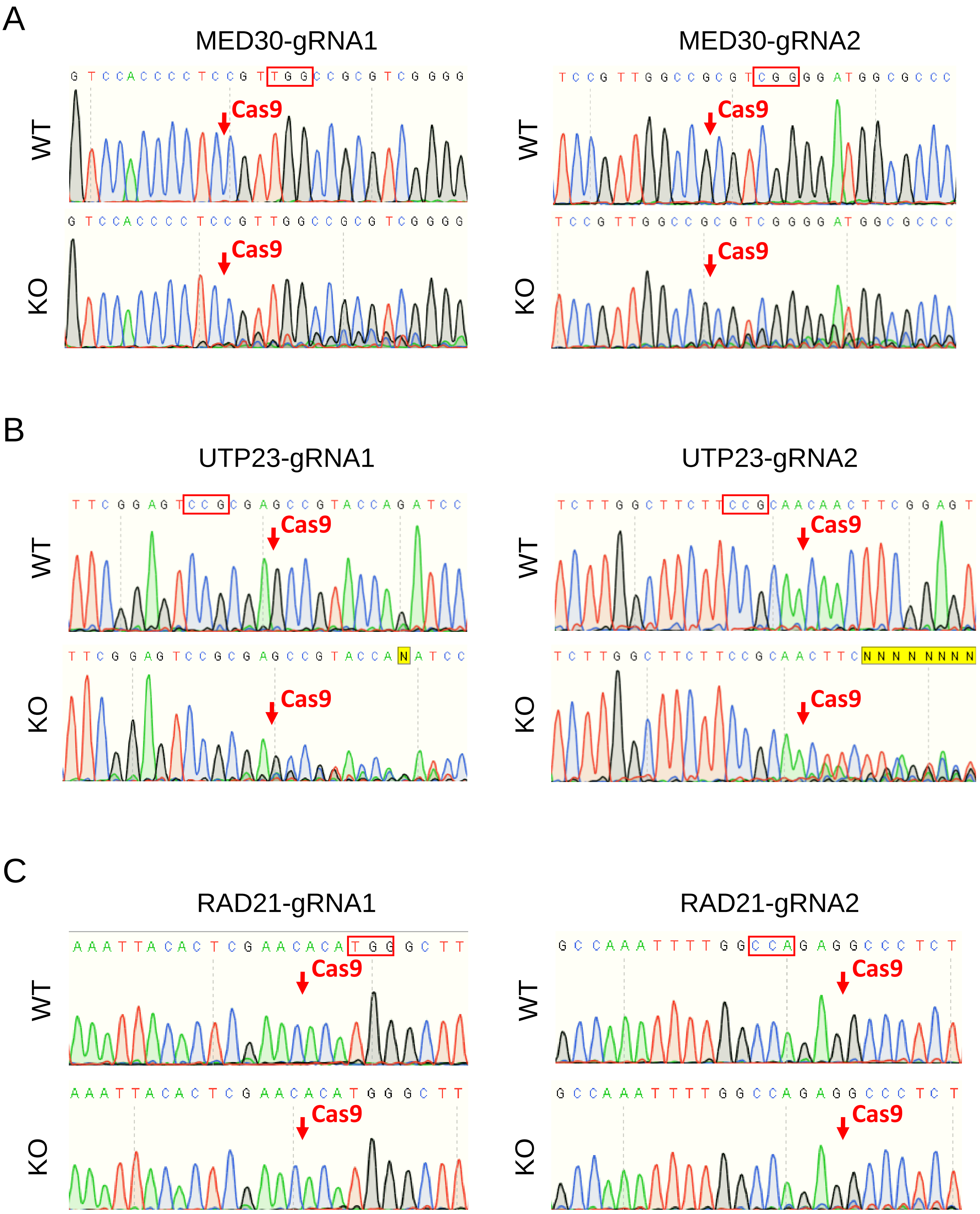
A
MED30-gRNA1
GTCCACCCCTCCGTTGGCCGCGTCGGGG
WT
Cas9
GTCCACCCCTCCGTTGGCCGCGTCGGGG
KO
Cas9
MED30-gRNA2
TCCGTTGGCCGCGTCGGGGATGGCGCCC
WT
Cas9
TCCGTTGGCCGCGTCGGGGATGGCGCCC
KO
Cas9
B
UTP23-gRNA1
TTCGGAGTCCGCGAGCCGTACCAGATCC
WT
Cas9
TTCGGAGTCCGCGAGCCGTACCANATCC
KO
Cas9
UTP23-gRNA2
TCTTGGCTTCTTCCGCAACAACTTCGGAGT
WT
Cas9
TCTTGGCTTCTTCCGCAACTTCNNNNNNNN
KO
Cas9
C
RAD21-gRNA1
AAATTACACTCGAACACATGGGCTT
WT
Cas9
AAATTACACTCGAACACATGGGCTT
KO
Cas9
RAD21-gRNA2
GCCAAATTTTGGCCAGAGGCCCTCT
WT
Cas9
GCCAAATTTTGGCCAGAGGCCCTCT
KO
Cas9

Figure S7

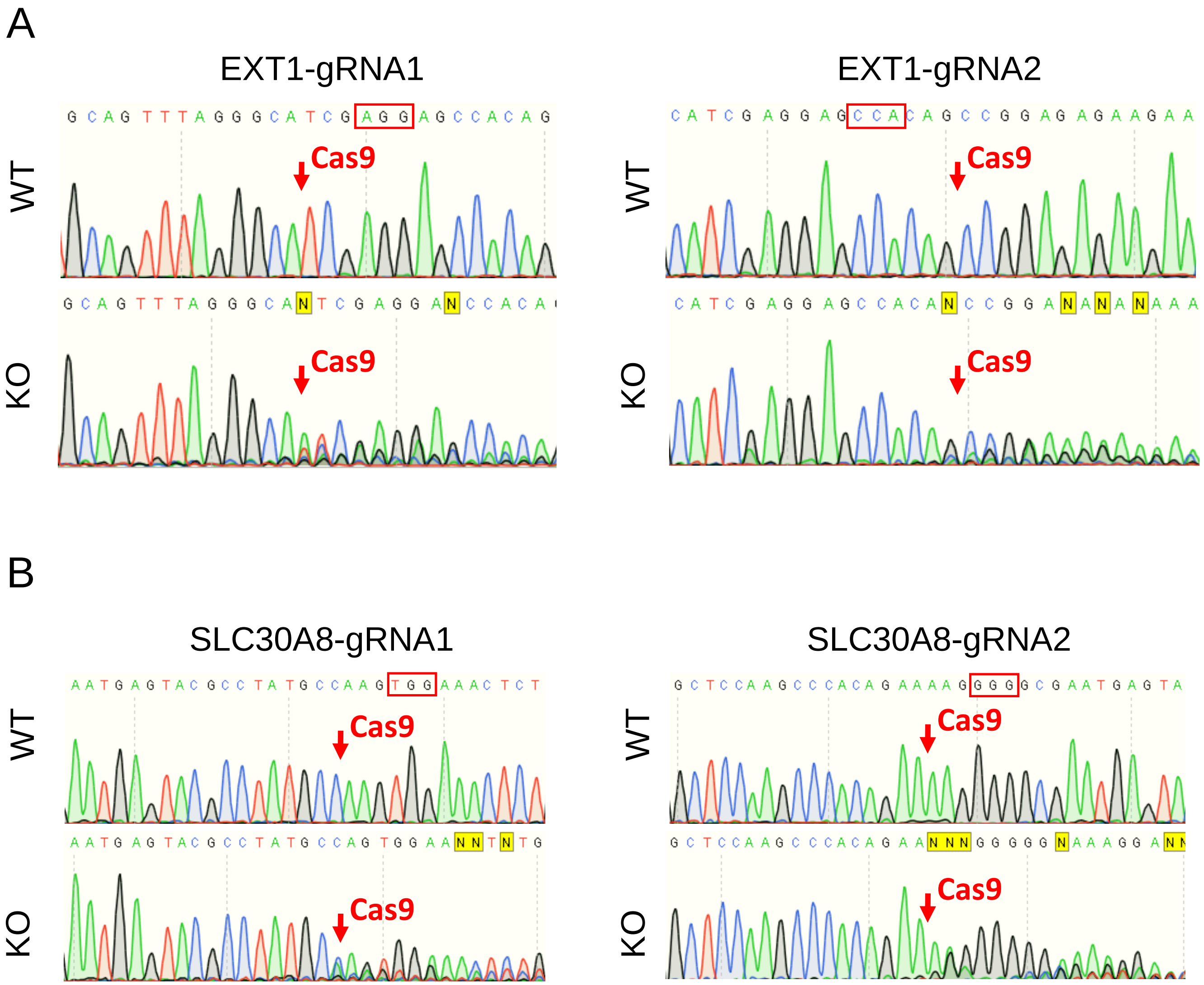
A
EXT1-gRNA1
EXT1-gRNA2
WT
KO
Cas9
B
SLC30A8-gRNA1
SLC30A8-gRNA2

Figure S8
